# Dual EZH1/2 inhibition enhances DNMT inhibitor efficacy in colon cancer through targeting H3K27me1

**DOI:** 10.1101/2025.09.16.676613

**Published:** 2025-09-18

**Authors:** Alison A. Chomiak, Ashley K. Wiseman, Joel Hrit, Yanqing Liu, Stephanie Stransky, Simone Sidoli, Rochelle L. Tiedemann, Scott B. Rothbart

**Affiliations:** 1 Department of Epigenetics, Van Andel Institute, Grand Rapids, Michigan 49503, USA.; 2 Van Andel Institute Graduate School, Grand Rapids, Michigan 49503, USA.; 3 Department of Biochemistry, Albert Einstein College of Medicine, Bronx, New York 10461, USA.

**Keywords:** DNA methylation, histone PTMs, EZH2, EZH1, H3K27me3, H3K27me1, H3K27ac, epigenetic therapy, colon cancer, DNA methylation inhibitors, decitabine, PRC2 inhibitors, tazemetostat, valemetostat, p300/CBP

## Abstract

Our recent work showed that low-dose DNMT inhibitor (DNMTi) treatment sensitizes colon cancer cells to EZH2 inhibitors (EZH2i), synergistically upregulating tumor suppressor genes (TSGs) and transposable elements through activation of the calcium-calcineurin-NFAT signaling pathway. A key observation was that EZH2i displayed variable sensitivities in combination therapy, which could not be explained solely by loss of lysine 27 trimethylation on histone H3 (H3K27me3), the most commonly studied EZH2 product. This led us to perform a comprehensive pharmacologic screen of Polycomb Repressive Complex 2 (PRC2) antagonists. Here, we show that compounds targeting both EZH2 and its interchangeable catalytic subunit, EZH1, achieved superior TSG re-expression when combined with DNMTi. Integrative proteomic and epigenomic analyses revealed that EZH1/2 inhibitors reduce all three H3K27 methylation states, whereas EZH2-selective inhibitors preserve EZH1-dependent H3K27me1 at deeply Polycomb-repressed genomic regions. Notably, H3K27me1 loss coincided with deposition of p300/CBP-dependent lysine 27 acetylation on histone H3 (H3K27ac), which proved essential for TSG re-expression. Paradoxically, blocking p300/CBP activity further enhanced the growth-inhibitory effects of combined DNMT and EZH1/2 inhibition. Mechanistically, we show that EZH1/2 inhibition redistributes p300/CBP activity, reducing H3K27ac from oncogenic loci and redirecting it to bivalent regions that enable TSG re-expression. Collectively, these findings reveal a coordinated role for EZH1-dependent H3K27me1 and DNA methylation in sustaining oncogenic transcriptional programs and provide strong rationale for advancing dual EZH1/2 inhibitors for combination epigenetic cancer therapy.

## Introduction

Focal DNA hypermethylation at promoter CpG islands along with global DNA hypomethylation are hallmarks of almost all cancers (1). DNA hypermethylation itself silences tumor suppressor genes and other loci implicated in almost every hallmark of cancer (2). Likewise, abnormal patterning of histone post-translational modifications (PTMs) dysregulates transcriptional profiles that contribute to oncogenesis (1,2). Despite the prevalence of these abnormal epigenetic patterns, there are few drugs that target DNA methylation or histone PTMs with demonstrated success in patients. These drugs include the nucleoside analogs azacytidine (AZA) and decitabine (DAC or 5-aza-2-deoxycytydine), which are pan-inhibitors of DNA methyltransferases (DNMT) 1, 3A, and 3B that reverse DNA hypermethylation. AZA and DAC both have U.S. Food and Drug Administration (FDA) approvals for use in blood malignancies and cancers (3-5). To target abnormal histone PTMs, several histone deacetylase inhibitors have received FDA approvals for liquid tumors (6,7). For solid tumors, the EZH2 methyltransferase inhibitor tazemetostat (TAZ or EPZ6438), which blocks Histone 3 Lysine 27 (H3K27) methylation, has first-in-class designations both as an FDA approved histone/lysine methyltransferase inhibitor and as an epigenetic therapy in rare solid tumors (8,9).

Limited drug exposure coupled with reduced replication rates in solid tumors compared to blood cancers may explain the limited successes of epigenetic therapy in this context. This presents a particular hurdle for nucleoside-analog DNMTi’s that require DNA replication for incorporation but have a short *in vivo* half-life coupled with dose-dependent DNA damage and toxicity (10,11). The recently FDA-approved and orally available ASTX727 (INQOVI) has been designed to circumvent this short half-life and increase tumor exposure. This formulation combines DAC and the cytidine deaminase inhibitor cedazuridine, which blocks the major *in vivo* elimination pathway of DAC (12). Additionally, attempts to avoid cytotoxicity associated with DNA incorporation have resulted in the development of the non-nucleoside, non-covalent, DNMT1-specific inhibitors GSK3484862 and GSK3685032 (13,14). The advent of these novel DNMTi formulations presents new opportunities for identifying mechanisms that could enhance DNMTi efficacy in solid tumors.

The plasticity of the epigenome and crosstalk between DNA methylation and histone PTMs represent such an opportunity for augmenting DNMTi therapy. We and others have shown that DNA hypomethylation can rewire the cancer epigenome and alter histone PTM patterning. In particular, hypomethylating the genome promotes compensatory layers of the repressive H3K27me3 along with crosstalk between H3K9me3 and H3K18ub that reinforce gene and transposable element (TE) silencing in many types of cancer (15-18). Altogether, this suggests that cancer cells remodel the epigenome as a survival mechanism, and this plasticity can be targeted/create a cancer vulnerability to design new combination treatments with DNMTi.

To identify targets that can augment DNMTi-induced reversal of gene silencing in colon cancer cells, we performed both a genome-wide siRNA screen and a comprehensive pharmacologic screen of antagonists of Polycomb Repressive Complex 2 (PRC2), which catalyzes all three methylation states of H3K27. We found genetically targeting SUZ12, which maintains PRC2 integrity, or chemically targeting both EZH2 and EZH1, the interchangeable PRC2 catalytic subunits, enhance transcriptional activation following DNMTi better than EZH2-specific loss or inhibition. Dual EZH1/2 inhibition reduces all three H3K27 methylation states, including EZH1-mediated H3K27me1 deposition. The loss of H3K27me1 and gain of H3K27ac at enhancers and regulatory regions supports p300-dependent gene activation when combined with DNA hypomethylation, while the loss of H3K27me1 and DNA methylation at genes bodies is associated with the downregulation of oncogenic transcriptional programs that drive cell growth. Consequently, colon cancer cells with high levels of EZH1 respond differentially to dual EZH1/2 and EZH2-selective inhibitors when combined with DNMTi. Altogether, this study reveals new findings for the role of EZH1-mediated H3K27me1 deposition in maintaining gene silencing while providing rationale for effective epigenetic combination therapies in colon cancer.

## Results

### The PRC2 subunit SUZ12 helps repress DNA methylation-silenced tumor suppressor gene

**Figure 1.**
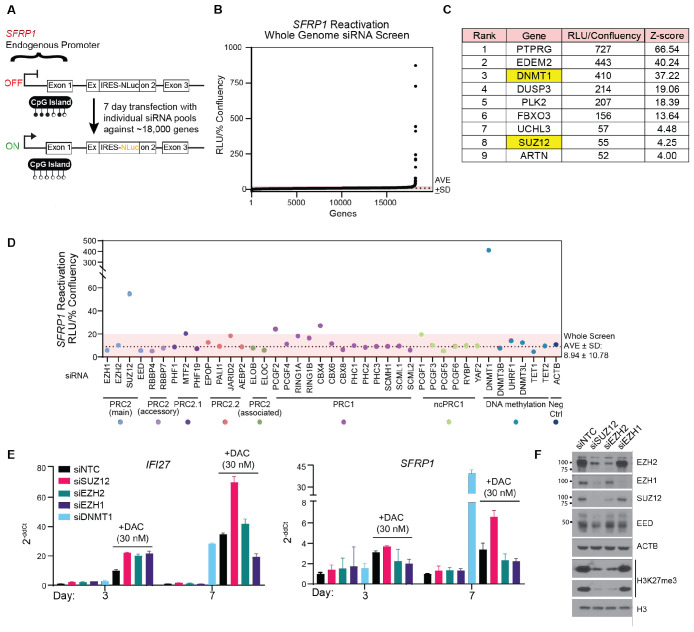
SUZ12 loss enhances transcriptional activating effects of DNA hypomethylation **A)** Approach for genome-wide siRNA screen using an endogenous *SFRP1* NanoLuciferase (NLuc) reporter cell line engineered in HCT116 cells with hypomorphic DNMT1. Closed circles represent methylated cytosines. Open circles represent potential demethylation following siRNA. **B)** Reactivation of the silenced *SFRP1* locus following siRNA-mediated knockdown of ~18,000 genes using the NLuc reporter in **A**. Relative luminescence units (RLU) from NLuc activity were normalized to cell confluency (%) per well for each siRNA pool. Results are ordered from lowest to highest normalized signal. Dotted line represents the screen average, and pink band represents ±1SD. **C)** Top gene hits with a Z-score >4 from **B** filtered for mRNA expression and confluency to avoid false positives and confounding toxicity, respectively. Yellow boxes are epigenetic or chromatin related genes. **D)** Select data points from **B** of genes related to PRC2, PRC1, or DNA methylation. Dotted line represents the screen average, and pink band represents ±1SD. **E)** Transcriptional validation by qRT-PCR of siDNMT1 and siSUZ12 responsive-gene targets (*IFI27* or *SFRP1*) following siRNA-mediated knockdown of *DNMT1*, *SUZ12,* or other PCR2 subunits with or without low-dose DAC (30 nM) for three or seven days in wild-type HCT116. Data are mean ±SEM of technical duplicates calculated using 2^−ΔΔC(t)^ normalized to both the housekeeping gene *RPL4* and siNTC (non-targeting control) samples and are representative of biological duplicate experiments. **F)** Western blot analysis of knockdown efficiency and H3K27me3 levels from wild-type HCT116 cells following three days of siRNA-mediated knockdown. **See also** Figure S1.

To identify genes that help maintain gene silencing following DNA hypomethylation, we performed a genome-wide siRNA genetic screen using a previously described HCT116 colon cancer cell reporter for the TSG *SFRP1* (Figure 1A) (15). Promoter CpG island hypermethylation flanked by H3K27me3 strongly silences *SFRP1*; Nanoluciferase (NLuc) activity from the NLuc cassette inserted downstream of the endogenous promoter reports on the reversal of *SFRP1* silencing whether through DNA hypomethylation and blocking H3K27me3 deposition or another mechanism (Figure 1A). This reporter cell line is engineered in a *DNMT1* hypomorph HCT116 derivative that reduces global DNA methylation ~20%, including at *SFRP1,* to increase amenability to high-throughput screening without the addition of DNMTi (19).

Using this reporter line, we individually transfected siRNA pools against ~18,000 genes over seven days, a timeframe optimized according to our previous report of *DNMT1* knockdown inducing NLuc activity in the NLuc reporter cell line (Figure 1A) (15). Following measurement of NLuc activity for each siRNA pool, we normalized the luminescence signal to a measure of confluency consistent with cell viability (Figure 1B and S1A). False positives based on basal state gene expression and knockdown of genes that inhibit cell viability rather than induce *SFRP1* expression were excluded to isolate regulatory effects on this silenced locus (Figure S1B-C, **Table S1**). From here we identified a limited list of gene targets whose knockdown contributes to *SFRP1* reactivation, including *DNMT1.* The only other epigenetic factor identified was *SUZ12,*, which encodes an indispensable scaffolding subunit for PRC2 activity (Figure 1C). We and others previously reported that PRC2 activity compensates to reinforce gene silencing following DNMT inhibition to maintain gene silencing in cancer, and this can be overcome through the use of inhibitors that selectively target EZH2, the primary catalytic subunit of PRC2 (15). As the screen surprisingly failed to identify *EZH2*, we compared *SUZ12* to all genes represented in the siRNA library and associated with PRC2, the complementary Polycomb Repressive Complex 1 (PRC1) that contributes to chromatin compaction/gene repression, and DNA methylation machinery. Indeed, only *SUZ12* and *DNMT1* knockdown robustly reactivated *SFRP1* (Figure 1D). The ability for SUZ12 but not EZH2 loss to induce *SFRP1* and other gene expression in a DNA hypomethylated state was validated by quantitative reverse transcription polymerase chain reaction (qRT-PCR) in wild-type HCT116 treated with low-dose DAC (Figure 1E and S1D).

We hypothesized that as a necessary/indispensable scaffold subunit for the core PRC2, *SUZ12* knockdown might show greater loss of the most commonly studied PRC2 methylation product, Histone 3 Lysine 27 trimethylation (H3K27me3) than *EZH2* knockdown. However, both *SUZ12* and *EZH2* knockdown, but not knockdown of the interchangeable catalytic subunit *EZH1*, showed similar robust efficacy at reducing H3K27me3 (Figure 1F). This indicated that levels of the repressive H3K27me3 cannot explain differences between SUZ12 and EZH2 in maintaining TSG silencing following DNA hypomethylation.

### Dual inhibition of EZH1 and EZH2 target all three H3K27 methylation states and enhances DNMTi-driven transcriptional upregulation

**Figure 2.**
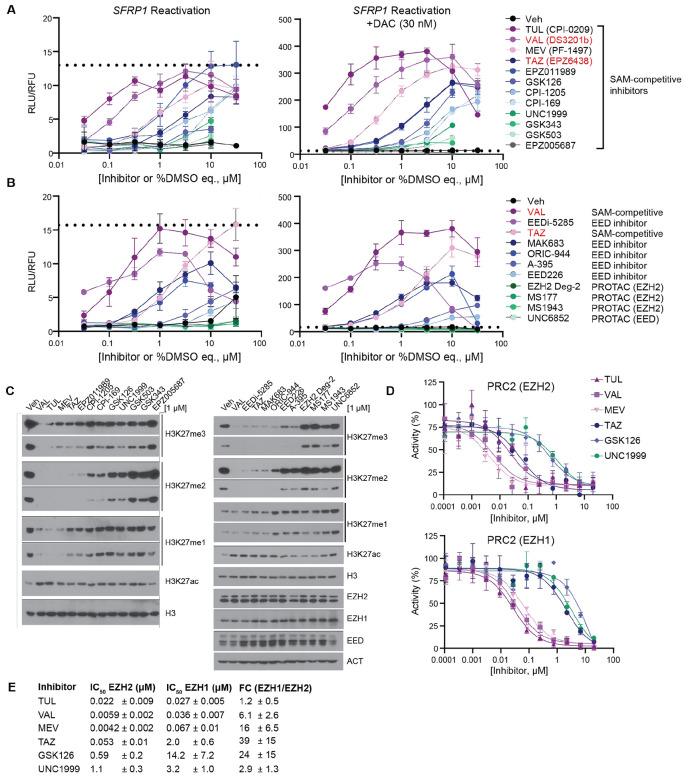
Dual EZH1/2 inhibitors block all H3K27 methylation states and enhance transcriptional activating effect of DNA hypomethylation **A-B)** NLuc reporter (Figure 1A) activity measurements following 72-hour treatment with the indicated SAM-competitive EZH inhibitors **(A)** or other PRC2 inhibitors, including EED inhibitors and EZH2 or EED PROTACs compared to VAL and TAZ (red) carried over from **A (B)**. Treatments are PRC2 inhibitors alone (left) or in combination with a fixed, low concentration of DAC (right). Relative luminescence units (RLU) are normalized to relative fluorescence units (RFU) from the CellTiter-Fluor cell viability assay. Data are mean ± SD of technical triplicates and are representative of three biological replicates. Dotted black lines denote scaling difference between left and right panels. TUL: tulmimetostat, VAL: valemetostat, MEV: mevrometostat, and TAZ: tazemetostat. **C)** Western blot analysis with short and long exposures of H3K27 modification states from wild-type HCT116 cells following 72-hour exposure to vehicle (DMSO, % equivalent) or PRC2 inhibitors (1 μM) ordered approximately by efficacy in **A-B**. **D)** *In vitro* methyltransferase activity assays with recombinant mononucleosome substrates and PRC2 methyltransferase complexes comprising core subunits SUZ12, EED, RbAp46/48 and either EZH2 or EZH1 as the catalytic subunit in the presence of selected SAM-competitive EZH inhibitors from panel **A**. Signal is normalized as percent of PRC2 activity in absence of inhibitor, and data are the mean ±SD of technical triplicates. **E)** IC_50_ values calculated from panel **D** and fold change (FC) of IC_50_ values for EZH1/EZH2. **See also** Figure S2.

As molecular measures of inhibition of H3K27me3 could not explain the insufficiency of *EZH2* knockdown at reactivating silenced loci, we performed a comprehensive pharmacologic screen of PRC2 antagonists to identify small molecules that phenocopy the transcriptional effect of *SUZ12* knockdown. This panel included experimental and clinically applied SAM-competitive inhibitors of EZH2 and EZH1, protein-protein interaction (PPI) disruptors of the indispensable PRC2 subunit EED and chromatin, and PROTAC degraders of PRC2 subunits. Three SAM-competitive molecules – Tulmimetostat (TUL or CPI-0209), Valemetostat (VAL or DS3201b), and Mevrometostat (MEV or PF-1497), showed superior efficacy at reactivating the *SFRP1-*NLuc reporter alone or with the addition of low-dose DAC compared to other SAM competitive molecules (Figure 2A). Additionally, VAL, representative of the top SAM-competitive molecules, outperformed all PPI disruptors and PROTAC degraders with or without low-dose DAC (Figure 2B). Consistent with our previous findings, PRC2 inhibitor efficacy in this assay did not correlate with toxicity (Figure S2A-B) (15). Notably, the superior PRC2 inhibitors have clinical approval in Japan (VAL) or have advanced to at least Phase II clinical trials in the U.S. (TUL and MEV), suggesting they share features that have contributed to their clinical advancement (20,21).

As PRC2 catalyzes all three H3K27 methylation states, we asked whether target inhibition outside of H3K27me3 explains the ability to synergize with DAC. Through western blot analysis, we found superior efficacy in synergizing with DAC correlates with PRC2 inhibitor efficacy in reducing not only H3K27me3, but also H3K27me2 and H3K27me1 along with increasing H3K27ac (Figure 2C and Figure S2C-D). In fact, H3K27me3 levels varied across PRC2 inhibitors, but the superior molecules – TUL, VAL, and MEV – robustly reduced all three methylation states, and this was particularly distinct for H3K27me1.

Both VAL and TUL are reported to be dual EZH1/2 inhibitors (EZH1/2i’s) (22,23). We wondered whether MEV also acts as a dual inhibitor and if this differentiates effective molecules from the other SAM-competitive inhibitors. To test this, we measured the ability of six representative molecules to inhibit the activity of either EZH1 or EZH2-containing PRC2 complexes toward recombinant nucleosomes in an *in vitro* methyltransferase assay. Consistent with our hypothesis, TUL, VAL, and MEV strongly inhibited EZH2-containing PRC2 in a dose-dependent manner and were also potent inhibitors of EZH1-containing PRC2 (Figure 2D). TAZ, which we previously reported synergizes with DAC (15) and which showed intermediate efficacy in cells (Figure 2A-B), was an effective inhibitor of PRC2 complexes containing EZH2 but not EZH1 (Figure 2D). GSK126 and UNC1999, which performed poorly in cells, were weak inhibitors of both types of PRC2 complexes. These data draw a correlation between dual potency against both EZH1 and EZH2 *in vitro* and efficacy at TSG reactivation and inhibition of all three H3K27 methylation states in cells. Collectively, these results suggest that EZH1 plays an important but overlooked role in H3K27 methylation states and gene repression.

### EZH1 contributes to the maintenance of H3K27 monomethylation when EZH2 is inhibited

**Figure 3.**
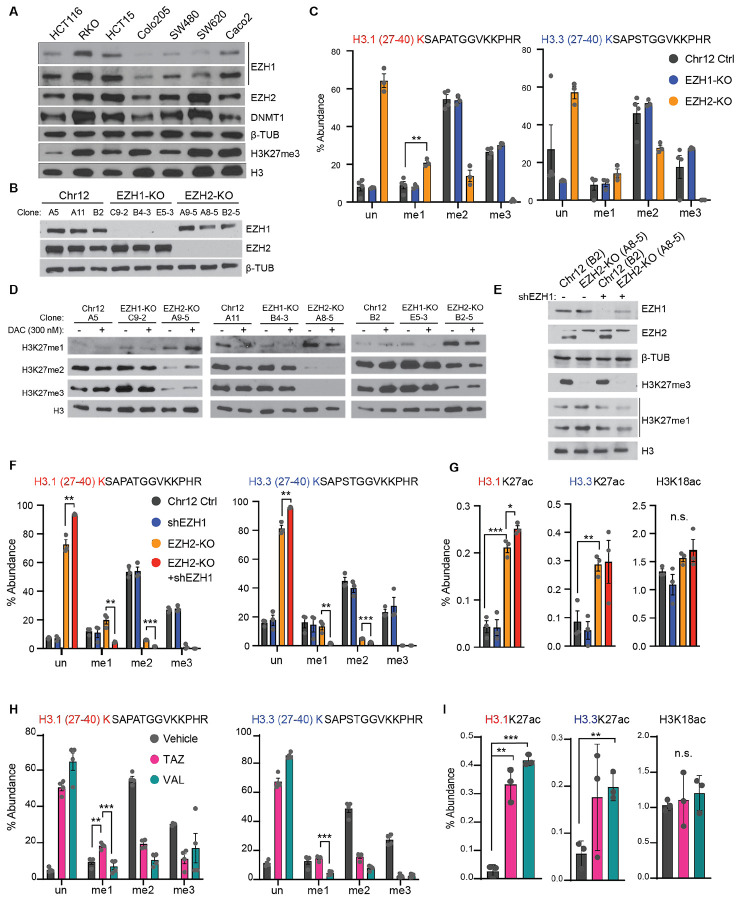
EZH1 contributes to H3K27me1 deposition in absence of EZH2 **A)** Immunoblot of EZH1 and EZH2 expression across human colon cancer cell lines. **B)** Immunoblot of EZH1 and EZH2 expression in EZH1 or EZH2 CRISPR knock out (KO) clones and Chr12 CRISPR control clones from RKO cells. Alphanumeric indicators represent three distinct clones selected for each control or KO line. **C)** Histone post-translational modification (PTM) mass spectrometry of relative abundances for H3K27 methylation states of H3.1 and H3.3 in individual RKO CRISPR clones from **B** represented by individual dots. Data are mean ± SD for three biological replicate clones for each CRISPR target. **D)** Immunoblot of the methylated states of H3K27 in RKO CRISPR KO clones treated with or without DAC at 300 nM for 3 days. **E)** Immunoblots for RKO Chr12 (clone B2) or EZH2-KO (clone A8-5) with induction of shEZH1 from 72 hours of doxycycline treatment. **F-G)** Histone PTM mass spectrometry relative abundances for **F)** H3K27 methylation states of H3.1 and H3.3 or **G)** acetylated histone peptides in RKO Chr12 or EZH2-KO clones with induction of shEZH1 by three days of doxycycline treatment. Data are mean ± SD for biological replicates each for clones in **E** with or without shEZH1. **H-I)** Histone PTM mass spectrometry relative abundances for **H)** H3K27 methylation states of H3.1 and H3.3 or **I)** acetylated histone peptides in RKO cells treated with the EZH2i tazemetostat (TAZ, 1 μM) or EZH1/2i valemetostat (VAL, 1 μM) for three days. Data are mean ± SD for four biological replicates. Statistical significance was calculated using multiple unpaired T-tests. *p<0.05, **p<0.01, ***p<0.001 **See also** Figure S3.

Although others have also recently shown the superiority of dual EZH1/2 inhibitors such as VAL, the contribution of EZH1 to H3K27 methylation states in cancer and the mechanism behind this superiority remains unclear (24,25). We hypothesized that EZH1 deposits H3K27me1 when EZH2 is inhibited, corresponding to EZH1’s reported role in monomethyl deposition (26,27). To test the contribution of EZH1 to H3K27 methylation state, we selected RKO out of a panel of colon cancer cell lines, as it has high expression of both EZH1 and EZH2 at the protein level (Figure 3A). Using CRISPR/Cas9, we targeted EZH1 and EZH2 for knockout (KO) and a gene desert on chromosome 12 (Chr12) as a CRISPR assay control. We isolated three clones for each CRISPR target and validated EZH1 or EZH2 loss (Figure 3B). We then measured global lysine methylation and acetylation at histone tails with mass spectrometry from these clones as a quantitative, antibody-independent approach to identify the contributions of each EZH methyltransferase to H3K27 methylation (Figure 3C and Figure S3A). EZH1-KO alone did not affect any of the methylation states of H3K27. EZH2-KO, however, significantly reduced levels of H3K27me2 and H3K27me3 on both histone variants H3.1 and H3.3 but surprisingly showed increased levels of H3K27me1 on the H3.1 peptide and maintained H3K27me1 on the H3.3 peptide. Notably, while H3.1 is replication coupled, H3.3 is replication-independent and primarily found in actively transcribed genes (28-30). We confirmed these results by immunoblot with and without the addition of the DNMTi DAC which showed no additional change (Figure 3D).

To determine if EZH1 is responsible for the increased H3K27me1 in the EZH2-KO cell lines, we introduced a dox-inducible shRNA for EZH1. This quickly and robustly leads to EZH1 knockdown and EZH2 knockout simultaneously while minimizing cell proliferation defects that come with a dual knockout. Indeed, knocking out EZH2 and knocking down EZH1 simultaneously reduced the level of H3K27me1 by both immunoblot and histone PTM mass spectrometry (Figure 3E-F, S3B). Interestingly, both EZH2-KO alone and EZH2-KO/EZH1 KD increased acetylation on H3K27 but not acetylation on other histone substrates (Figure 1G).

We next asked if the EZH2-selective inhibitor TAZ (Figure 2) phenocopies EZH2-KO and if the dual EZH1/2 inhibitor VAL phenocopies dual EZH2-KO/EZH1-KD cells. These molecules have US FDA and Japan Ministry of Health, Labor and Welfare approvals, respectively, and are both actively used clinically. As we observed with the EZH2-KO clones, cells treated with TAZ had greatly reduced levels of H3K27me2 and H3K27me3 but had increased levels of H3K27me1 (Figure 3H, Figure S3C). Similarly, as we saw with the EZH2-KO/shEZH1 cell line, we observed a much stronger reduction in the H3K27me2 and H3K27me3 marks after VAL treatment, as well as a decrease in H3K27me1 on both H3.1 and H3.3. These trends also repeated when combining TAZ or VAL with the DNMTi DAC (Figure S3D). Both genetic manipulations with CRISPR EZH2-KO and shEZH1 and pharmacologically with TAZ or VAL treatments increased the abundance of the H3K27ac peptide, a target of the histone acetyl transferases (HATs) p300/CBP, while the other known p300/CBP-dependent acetylation target, H3K18, and other histone acetyl PTMs remain unchanged (Figure 3G,I and S3E), indicating a potential relationship between this increase in acetylation and the decreasing methylation levels at H3K27 following TAZ or VAL treatment.

### Underlying genomic distributions of H3K27me3 and DNA methylation shape H3K27me1 patterning and response to EZHi/DNMTi treatment

Mass spectrometry data suggested the most unique histone PTM change was H3K27me1 depletion following EZH1/2 loss or dual EZH1/2 inhibition. This prompted us to ask where changes to H3K27me1 occur in the genome. Given the limited number of studies profiling H3K27me1 in cancer, we first validated H3K27me1 antibodies before proceeding with our quantitative ChIP-seq method, siQ-ChIP (31,32), to answer this question (Figure S4A). Using our histone peptide array, we identified a highly specific H3K27me1 antibody from ActiveMotif (61015) whose antibody:chromatin isotherm indicated antibody titrations could capture chromatin mass in accordance with the histone PTM mass spectrometry profiles following VAL or TAZ treatments (Figure S4B). Importantly, other H3K27me1 antibodies (Invitrogen and Abclonal) showed either high variation between lots and/or off-target activity against high abundance PTMs such as H3K9me1 (Figure S4C-D). Using our validated antibody, we profiled H3K27me1 distributions with quantitative resolution to establish the baseline pattern and response of this modification to TAZ or VAL treatments.

Consistent with reports in mouse embryonic and human hematopoietic stem cells, H3K27me1 distributions in vehicle (VEH)-treated cells were primarily enriched across gene bodies of transcribed genes (Figure S5A) (33,34). Using our previously published and antibody-validated ChIP-seq data for H3K27me3 (Figure S4E) and publicly available whole genome bisulfite sequencing (WGBS) data for 5mC (16,35), we profiled the underlying baseline distribution of these primary epigenetic marks targeted by our drug combination. At baseline, enriched H3K27me1 profiles overlap with highly DNA methylated domains where H3K27me3 is notably absent (Figure S5B), an epigenetic pattern consistent with transcribed gene bodies (Figure S5A) (33).

**Figure 4.**
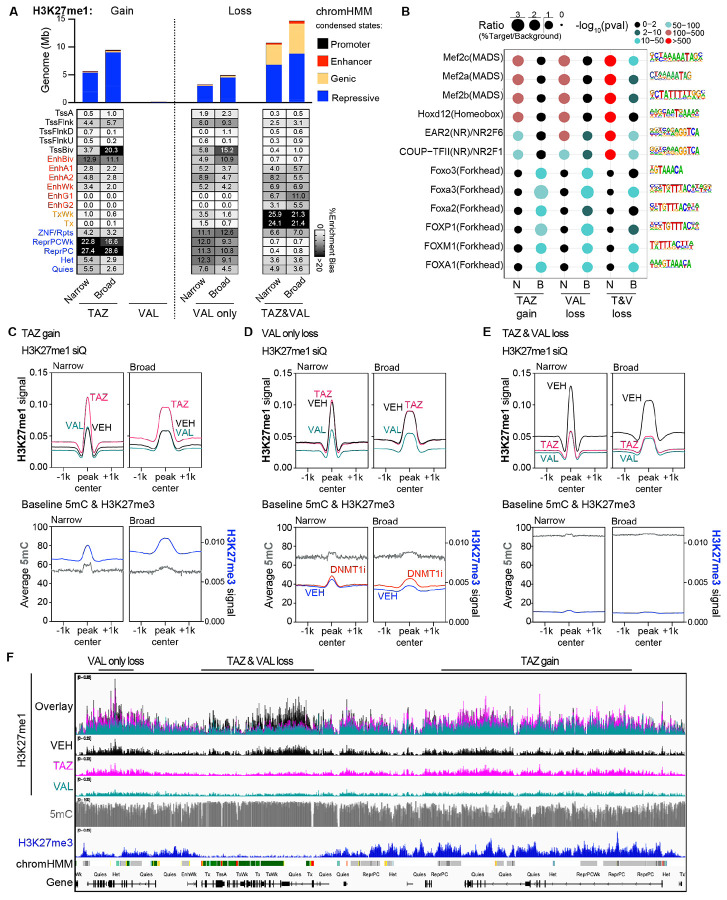
The underlying genomic distributions of H3K27me3 and DNA methylation shape H3K27me1 patterning and response to EZHi/DNMTi treatment **A)** chromHMM characterization of EZHi-associated significantly altered regions of H3K27me1 efficiency (siQ-ChIP, n=3 biological replicates) relative to VEH (% eq., DMSO) treated RKO colorectal cancer cells. Top: Genomic coverage (in Mbs) of H3K27me1 gain (left) and H3K27me1 loss (right) induced by EZHi. Bottom: Relative enrichment bias for altered H3K27me1 distributions across chromHMM states. **B)** HOMER motif enrichment analysis for EZHi-associated H3K27me1 gains and loss described in **A**. Size of the bubble represents the ratio of (%H3K27me1 change with motif target)/(%Background with motif target); the larger the bubble the greater the enrichment above background. Bubble color represents significance of the enrichment. **C-E)** Average epigenomic profiles for H3K27me1 (top) and baseline 5mC (left y-axis) and H3K27me3 (right y-axis) signals (bottom) for (C)TAZ-associated H3K27me1 gains, (D) VAL-only associated H3K27me1 losses, and (E) EZHi-associated H3K27me1 losses shared among TAZ and VAL treatment as defined in **A**. **F)** Representative browser shot of epigenomic patterning described. All drug treatments were conducted for 72 hours in RKO cells. Drug concentrations: TAZ (1 μM), VAL (1 μM), and DAC (100 nM). **See also** Figure S4-5.

We next quantified the change in H3K27me1 distribution from these baseline patterns following TAZ or VAL treatments. For both H3K27me1 gains and losses induced by drug treatments, we observed a high density of relatively short peaks (~300-400 bp) and a tail of broader peaks classified using Gaussian Mixture Modeling (Figure S5C). Consistent with the mass spectrometry, siQ-ChIP showed gains of H3K27me1 signal with TAZ treatment alone, primarily occurring in repressive, polycomb marked regions of the genome, whereas VAL induced minimal gains of H3K27me1 (Figure 4A). Narrow TAZ-associated H3K27me1 gains were additionally present at bivalent enhancers (EnhBiv) while broad peaks were additionally localized at bivalent promoters (TssBiv). We also identified regions that maintained H3K27me1 with TAZ treatment but only lost this modification with VAL (Figure 4A). Importantly, the enrichment for VAL only H3K27me1 loss occurred at similar chromHMM states as the TAZ-associated H3K27me1 gains, indicating potential similarity in the epigenetic regulation of these regions. Finally, H3K27me1 loss occurring in both TAZ and VAL treatments was enriched for transcribed gene bodies and enhancer regions (Figure 4A).

Given the noted enrichment for H3K27me1 changes at both enhancers and promoters, we next asked if particular motifs were found at regions of EZHi-altered H3K27me1. Consistent with shared chromHMM state patterns, narrow peaks of TAZ-associated H3K27me1 gain and VAL only H3K27me1 loss demonstrated highly significant enrichment for Mef2 and Hox developmental transcription factor binding motifs, with over 25% of TAZ-associated H3K27me1 gains predicted to be Mef2 targets (Figure 4B). Broad peaks of TAZ-associated H3K27me1 gain and VAL only H3K27me1 loss were significantly enriched for FOX transcription factor binding motifs (Figure 4B). Finally, H3K27me1 loss that both TAZ and VAL treatment share was additionally enriched for Mef2, Hoxd, and NR2F at narrow peaks and FOX transcription factors at broad peaks (Figure 4B). Given the specific and consistent enrichment for chromHMM states and developmentally associated TF binding motifs among H3K27me1 changes, these results suggest that H3K27me1 holds an important role at these specific regulatory regions. Indeed, EZH1 is required for cardiac regeneration and proper hematopoietic differentiation while EZH2 is dispensable, indicating that EZH1 (and presumably its ability to maintain H3K27me1 at these regions (Figure 4A)), plays important roles for gene regulation and cellular differentiation (Figure S5D) (36-39).

To understand the underlying epigenetic regulation of these regions where H3K27me1 changes, we again profiled baseline H3K27me3 and 5mC centered at these altered H3K27me1 peaks, and we found the baseline epigenetic patterns differed between the categories of H3K27me1 gain or loss in Figure 4A. TAZ-associated H3K27me1 gained peaks had relatively low baseline levels of H3K27me1 and partial DNA methylation yet high baseline levels of H3K27me3, indicative of deep polycomb repression (Figure 4C and S5D). The shape of these TAZ-associated H3K27me1 gains mirror that of the baseline H3K27me3 distribution, which could suggest a failure to catalyze up to trimethylation when EZH2 is inhibited (Figure 4C). DNA methylation patterning in these regions reflects that of deep polycomb repression with partial DNA methylation profiles (Figure 4C, S5D). In comparison, regions susceptible to VAL only-induced H3K27me1 loss demonstrated a distinct underlying profile of H3K27me3 and 5mC patterning (Figure 4D). Instead, the baseline level (VEH-treated) of both H3K27me1 and 5mC are higher at the peak whereas the H3K27me3 signal is lower in these regions (Figure 4D and S5D). This occurred both in repressive polycomb regions but more broadly in other repressive chromHMM states as well (ZNF/Rpts, Het) that demonstrate a potential “epigenetic switch” between DNA methylation and H3K27me3 when challenged by DNMT1 inhibition (Figure 4A and 4D). Finally, H3K27me1 loss that occurs with both TAZ and VAL treatment overlaps significantly with high H3K27me1 signals observed in VEH (Figure S5A), correlating with highly DNA methylated regions and absent H3K27me3 and consistent with the strong associations we observed between H3K27me1 and 5mC at actively transcribed regions (Figure 4E, S5A, D).

Collectively, these data demonstrate the underlying profile of H3K27me3 largely dictate the H3K27me1 distributions and response to TAZ or VAL (Figure 4F). When H3K27me3 is high and baseline H3K27me1 levels are low, VAL efficiently depletes all three methylation states, while TAZ treatment induces a gain of H3K27me1 at these deep polycomb marked regions (Figure 4F). This suggests that in the absence of EZH2 (and associated H3K27me3 loss), EZH1 targets these regions for H3K27me1 deposition. While having similar chromHMM state and motif enrichment profiles as TAZ-associated H3K27me1 gains, VAL only H3K27me1 loss occurs at genomic regions with a mix of intermediate H3K27me1, 5mC, and H3K27me3 profiles, potentially indicating these regions are more heterogenous in their epigenetic patterning in our cell population and more vulnerable to epigenetic therapy targeting both EZH1 and EZH2 (Figure 4F). Finally, we observed that H3K27me1 and DNA methylation highly correlate across actively transcribed genes, and both TAZ and VAL deplete H3K27me1 similarly in these regions (Figure 4F). Like DNA methylation, H3K27me1 distributions are found in both repressive and active regions of the genome, with TAZ and VAL treatments modulating H3K27me1 in context-specific ways.

### EZHi/DNMTi Combination Therapy Redistributes H3K27ac to Promote Transcriptional Activation

**Figure 5.**
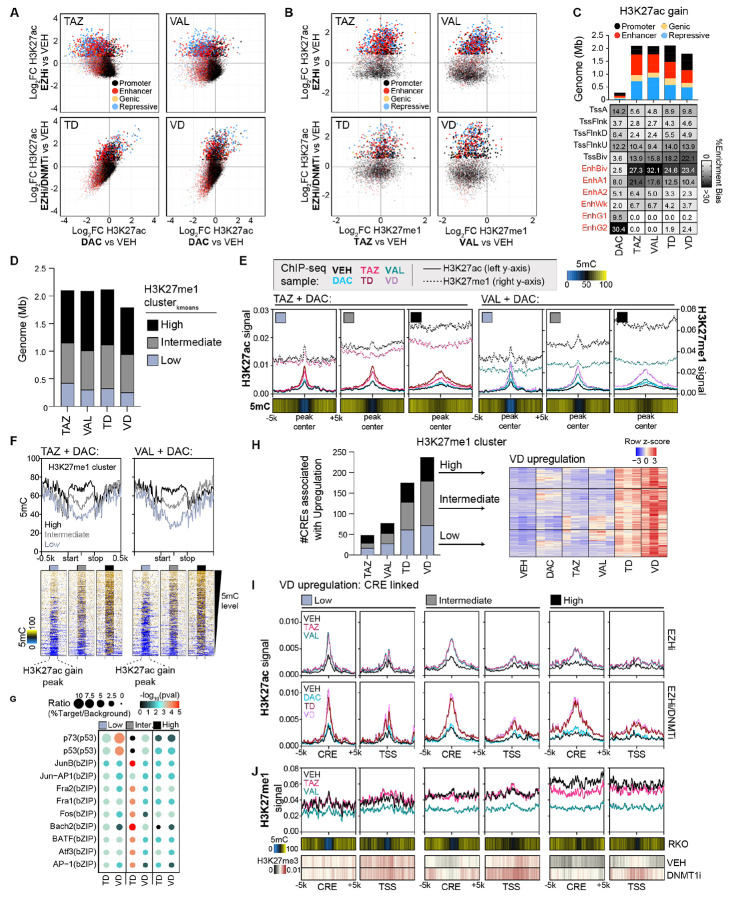
Combination EZHi/DNMTi redistributes H3K27ac to bivalent genes and promotes transcriptional activation. **A)** Scatterplot of H3K27ac change following single-agent DNMTi (DAC) treatment (x-axis) compared to the H3K27ac change in single-agent EZHi (top row) and combination DNMTi/EZHi (bottom row) treatments (y-axis). H3K27ac peaks are colored by their condensed chromHMM state. Large dots represent significant H3K27ac gains (log_2_FC ≥ 0.585) relative to VEH. **B)** Scatterplot of H3K27me1 change in single-agent EZHi treatment (x-axis) compared to the H3K27ac change in single-agent EZHi (top row) and combination DNMTi/EZHi (bottom row) treatments for H3K27ac gains that occurred within 10kb (+/−) of H3K27me1 signal. H3K27ac peaks are colored by their condensed chromHMM state. Large dots represent significant H3K27ac gains of ≥ 1.5 fold (log_2_FC ≥ 0.585) relative to VEH. **C)** chromHMM characterization (condensed states) of significant H3K27ac gains described in **B**. Top: Stacked bar graph of H3K27ac gains in genomic coverage (Mbs). Bottom: Relative enrichment bias for altered H3K27ac gained distributions across chromHMM states. **D)** Stacked bar graph of H3K27ac gains genomic coverage (Mbs) clustered by H3K27me1 signal (kmeans = 3). **E)** Epigenetic profiling of H3K27ac gains (from **B**) in EZHi/DNMTi treatments, clustered by H3K27me1. Top: Average H3K27ac (left y-axis) and H3K27me1 (right y-axis) siQ-ChIP efficiency centered on EZHi/DNMTi associated H3K27ac gains. Bottom: Baseline average DNA methylation in RKO cells centered on H3K27ac gains. **F)** DNA methylation analysis within scaled H3K27ac gained peaks (from **B**) in EZHi/DNMTi treatments, clustered by H3K27me1. Top: Average DNA methylation profiles (WGBS) in parental RKO cells (GSE262054). Bottom: Heatmap of DNA methylation (WGBS) across individual H3K27ac gained peaks, sorted from the peak with the highest DNA methylation level to the lowest. **G)** HOMER motif enrichment analysis for EZHi/DNMTi associated H3K27ac gains clustered by H3K27me1 levels (from **B**). Size of the bubble represents the ratio of (%H3K27ac gains with motif target)/(%Background with motif target), the larger the bubble the more enrichment above background. Bubble color represents significance of the enrichment. **H)** VD-associated H3K27ac gains at CREs induce transcriptional upregulation of distal, bivalent genes. Left: Number of cis-regulatory element (CRE) H3K27ac gains associated with transcriptional upregulation induced by respective EZHi and EZHi/DNMTi treatments, clustered by H3K27me1 signal. Right: Heatmap of gene expression across drug treatments from respective VD H3K27me1 clusters from left. **I)** Average H3K27ac siq-ChIP efficiency centered on the CRE-TSS VD-upregulated genes described in **H**, clustered by H3K27me1. Top row: single-agent EZHi treatments, bottom row: EZHi/DNMTi treatments. **J)** Epigenetic profiling and loci characterization of regions described in **I**. Top: Average H3K27me1 siQ-ChIP efficiency profiles, middle: baseline DNA methylation (GSE262054), and bottom: baseline and DNMT1i (GSK3484862) H3K27me3 signal, centered on linked CRE-TSS VD upregulated gene pairs. TD: TAZ + DAC, VD: VAL + DAC. **See also** Figure S6-7.

Mass spectrometry analysis additionally revealed that EZHi treatments result in H3K27ac increases. We confirmed the H3K27ac antibody specificity (Figure S4F) and used quantitative H3K27ac siQ-ChIP to profile the distribution of H3K27ac changes with single-agent EZHi or EZHi/DNMTi drug treatments. EZHi single-agent and combination treatments all induced dynamic H3K27ac changes of not only gain but also loss (Figure S6A). Gains occurred at genomic locations with low basal H3K27ac level and loss at locations with high basal H3K27ac levels (Figure S6A). Importantly, while DNMTi treatment alone had marginal effects on H3K27ac, the addition of DNMTi to EZHi treatment not only enhanced the H3K27ac gains and losses (Figure S6A) but also shifted the genomic distribution of these changes relative to EZHi single-agent treatments (Figure 5A). We hypothesized that EZH1/2i/DNMTi (VD) treatment efficiently reduces all H3K27 methylation states and DNA methylation to allow for p300/CBP deposition of H3K27ac and gene transcription activation. To evaluate this hypothesis, we first focused on the H3K27ac gain induced across our EZHi or EZHi/DNMTi drug treatments that occurred within 10 kilobases (upstream or downstream) of H3K27me1 marks (Figure 5B). While VAL-associated H3K27ac gains almost exclusively coincided with H3K27me1 loss, TAZ-associated H3K27ac gains occurred both at regions with H3K27me1 loss and gain (Figure 5B). Single-agent EZHi treatment induced H3K27ac gains primarily at enhancers and repressive regions of the genome (Figure 5B,C). The addition of DNMTi to EZHi treatments shifted H3K27ac gains to different genomic coordinates than occurred with single-agent EZHi (Figure S6B) and enhanced H3K27ac gains at promoter regions (Figure 5C). Collectively, these results indicate that although single-agent DNMTi treatment minimally impacts overall H3K27ac gain, it substantially influences the genomic redistribution of H3K27ac in the context of EZHi combination treatment.

We next sought to understand how H3K27me1 modulation integrates with EZHi/DNMTi induced H3K27ac gain given their shared substrate. We clustered H3K27ac gains across our drug treatment paradigms individually by the baseline H3K27me1 signal (Figure 5D). H3K27me1 clustering of H3K27ac gains revealed a divergence for enrichment in chromHMM states between single-agent EZHi and EZHi/DNMTi treatment (Figure S6C). While single-agent EZHi treatment consistently enriched for H3K27ac gain at enhancers across all three H3K27me1 clusters, EZHi/DNMTi treatment enriched for H3K27ac gain at promoters (including TssBiv) in the low cluster, both promoters and enhancers in the intermediate cluster, and bivalent enhancers in the high cluster (Figure S6C). For single-agent EZHi treatments, we consistently observed among the three different H3K27me1 clusters that DAC treatment has no effect on H3K27ac at these regions (Figure S6D) despite the high levels of DNA methylation present at these regions. Indeed, single-agent EZHi treatments induced a H3K27ac/5mC bivalent state at these enhancers across all H3K27me1 clusters (Figure S6E). In contrast, DAC treatment alone induces small yet measurable increases in H3K27ac signal at EZHi/DNMTi H3K27ac gains, and indeed, appears to synergize with EZHi to amplify the H3K27ac signal at these regions (Figure 5E). Unlike single-agent EZHi treatments, H3K27ac gains induced by combination EZHi/DNMTi treatment demonstrated a baseline DNA methylation status that correlates with H3K27me1 signal (Figure 5F and S6E); the higher the H3K27me1 signal the higher the DNA methylation level within the gained H3K27ac peak. In all cases, across all EZHi treatments and H3K27me1 clusters, on average, loss of H3K27me1 coincides with H3K27ac gain, suggesting that the reduction in H3K27 monomethyl states do indeed provide an opportunity for H3K27ac acquisition (Figure 5E and S6D).

EZHi/DNMTi treatments demonstrated particularly strong enrichment for p53/p73 and AP-1 (Jun/Fos) transcription factor motifs in the low and intermediate 5mC/H3K27me1 clusters, respectively (Figure 5G). Notably, p73 and AP-1 transcription factors have previously been shown to share target genes that regulate the apoptosis-survival balance of cells, and our prior work showed that DAC treatment induced an “epigenetic” switch from DNA methylation to H3K27me3 repression at AP-1 binding motifs (15,40). To determine if H3K27ac gains influence gene transcription, we used Genomic Regions Enrichment of Annotations Tool (GREAT) to link H3K27ac gains at cis-regulatory elements (CREs) to differentially upregulated genes induced by our drug combinations (Figure 5H) (41). Importantly, EZHi/DNMTi-induced H3K27ac gains at CREs were more tightly linked to transcriptional upregulation than EZHi alone (Figure 5H).

To understand the epigenetic regulation of this CRE-linked VAL+DAC (VD) transcriptional upregulation, we next profiled the H3K27ac (Figure 5I) and H3K27me1 (Figure 5J) dynamics at the CRE and linked TSS among our H3K27me1 clusters. In the low H3K27me1 cluster, both the CRE and linked TSS demonstrate synergistic H3K27ac gain by DAC treatment with both TAZ and VAL (Figure 5I). However, H3K27me1 dynamics at these genes are not shared between single-agent EZHi treatment as VAL effectively depletes H3K27me1 at both the CRE and TSS while TAZ mildly reduces H3K27me1 at the CRE-center and does not change H3K27me1 at the TSS (Figure 5J and S7A). Indeed, both the CRE and TSS of the low H3K27me1 clustered genes demonstrate baseline H3K27me3 that does not change when challenged by DNMT1i (Figure 5J). These results suggest that VAL use in EZHi/DNMTi combination treatment is superior to TAZ at activating these genes due to dual targeting of EZH1/2 and reduction of H3K27me1 at both the CRE and TSS.

In the H3K27me1 intermediate cluster, we again observed synergy of DAC with both TAZ and VAL for H3K27ac gains at both the CRE and TSS (Figure 5I and S7A). Like the H3K27me1 low cluster, we observed that H3K27me1 is mildly reduced at the CRE and does not change at the TSS with TAZ treatment while VAL depleted H3K27me1 at both regions. Significantly, the TSS of these genes are marked by DNA methylation, and prior work from our group demonstrated that these TSSs are prone to an “epigenetic switch” from DNA methylation to H3K27me3 when challenged by DNMTi alone (Figure 5I). We hypothesized that the synergy for H3K27ac gain at these TSSs occurs from a block in the “epigenetic switch” by combination EZHi/DNMTi, and indeed we observed that both CRE and TSSs within this cluster demonstrate a gain in H3K27me3 when challenged with DNMT1i (Figure 5J). Finally, the high 5mC/H3K27me1 cluster demonstrates synergistic/additive H3K27ac gain at CREs and TSSs for both TAZ and VAL in combination with DAC (Figure 5I and S7A), and H3K27me1 is reduced by TAZ and depleted by VAL (Figure 5J). Taken together, we conclude that the addition of DNMTi to EZHi treatment redistributes H3K27ac profiles to mediate transcriptional activation of bivalent epigenetically suppressed gene pathways.

### Combination EZHi/DNMTi treatment represses oncogenic transcriptional programs independently of H3K27ac driven gene activation

**Figure 6.**
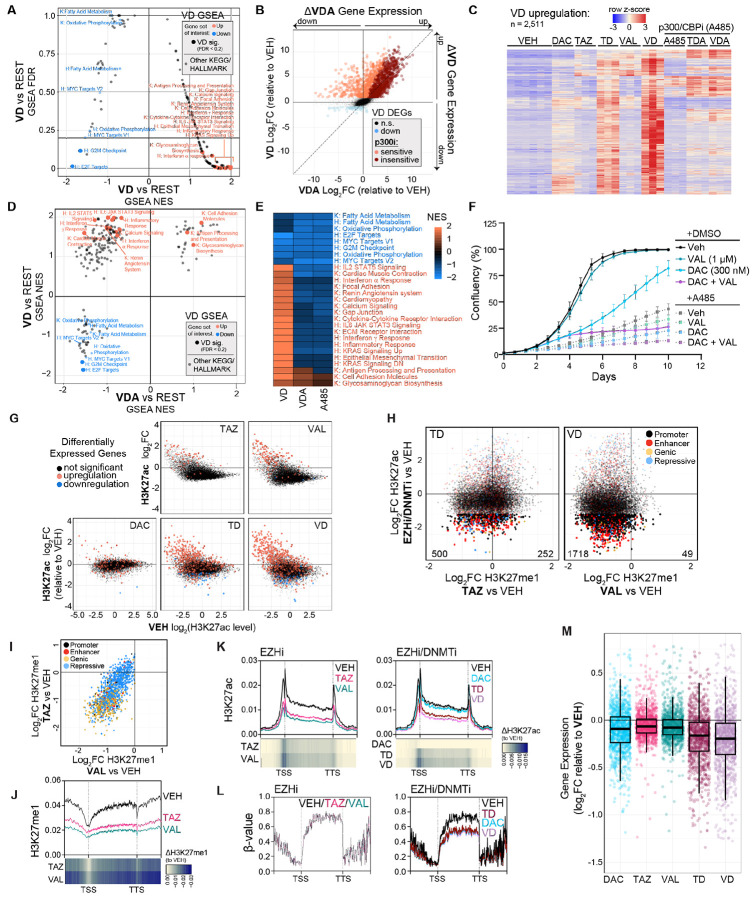
Combination EZHi/DNMTi represses oncogenic programs independently of H3K27ac driven gene activation **A)** Gene set enrichment analysis (GSEA) for VD (VAL+DAC)-associated transcription versus REST (VEH/DAC/VAL) across HALLMARK and KEGG gene sets. **B)** Scatterplot of gene expression change (relative to VEH) between VD treatment (y-axis) versus VD + the p300/CBP inhibitor A485 (VDA) treatment (x-axis). Each dot represents an individual gene with coordinates derived from designated drug treatment gene expression change (Log_2_FC relative to VEH). **C)** Supervised clustered heatmap of relative gene expression across drug treatments (row z-score, each row is a gene) for genes significantly upregulated by VD treatment. Roughly 83% of genes are sensitive to A485 treatment. **D)** Scatterplot of GSEA results between VD treatment (y-axis) versus VDA treatment (x-axis). Each dot represents an individual gene set Normalized Enrichment Score (NES) for the designated drug treatment within the HALLMARK/KEGG gene sets. **E)** Supervised clustered heatmap of NES across drug treatments for gene sets of interest. **F)** Outgrowth measurements (% confluency) of RKO cells treated once with DAC (300 nM) and/or valemetostat (VAL, 1 μM) with or without A485 (10 μM) and observed for 10 days. Data are the mean ±SEM of technical replicates from a single experiment (n=12 images per timepoint and treatment) and are representative of three biological replicates. **G)** MA plots for level of H3K27ac (in VEH) versus change in H3K27ac signal (relative to VEH) across the drug treatments. H3K27ac peaks within 10kb of a gene promoter were considered. Differentially expressed genes for each drug treatment are highlighted for their association with H3K27ac dynamics. **H)** Scatterplot of H3K27me1 change in single-agent EZHi treatment (x-axis) compared to the H3K27ac change in EZHi/DNMTi combinations treatments for H3K27ac loss that occurred within 20kb (+/−) of H3K27me1 signal. H3K27ac peaks are colored by their condensed chromHMM state. Large dots represent significant H3K27ac loss with FC ≤ 0.5 (log_2_FC ≤ −1) relative to VEH. **I)** Scatterplot of H3K27me1 change (within 20 kb of H3K27ac loss) between single-agent EZH1/2i (VAL) and EZH2i (TAZ) treatments at VD-associated significant H3K27ac loss (bottom left quadrant of VD plot in **H**). H3K27ac peaks are colored by the condensed chromHMM state of the nearest H3K27me1 peak. **J)** Average profiles of gene body H3K27me1 signal (siQ-ChIP) from genes described in **I**. The average profiles are presented on top with the change in the modification relative to VEH presented as heatmaps on bottom. **K)** Average profiles of gene body H3K27ac signal (siQ-ChIP) from genes described in **I** for single-agent EZHi (left) and EZHi/DNMTi (right). The average profiles are presented on top with the change in the modification relative to VEH presented as heatmaps on bottom. **L)** Average profiles of gene body DNA methylation (EPIC array) from genes described in **I** for single-agent EZHi (left) and EZHi/DNMTi (right) The average profiles are presented on top with the change in the modification relative to VEH presented as heatmaps on bottom. **M)** Boxplot of the change in gene expression (relative to VEH) for each drug treatment for genes described in **I**. Dots represent individual genes. **See also** Figure S8.

Consistent with our previous study (15), VAL in combination with DAC primarily upregulated pro-inflammatory, immune response, and calcium signaling pathways (Figure 6A). Given the H3K27ac acquisition at CREs and bivalent promoters observed following EZHi/DNMTi and associated with transcriptional upregulation (Figure 5), we asked whether these transcriptional activating effects depend on p300/CBP. Using a concentration of the p300/CBP inhibitor A485 that maximizes target inhibition when combined with EZHi’s while minimizing toxicity (Figure S8A-C), we observed attenuation of over 80% of EZHi/DNMTi upregulated genes (Figure 6B,C) and the aforementioned VD upregulated GSEA gene sets (Figure 6D, E). Likewise, A485 showed dose-dependent inhibition of *SFRP1* reactivation when combined with either EZHi and DAC in the HCT116 NLuc cell reporter (Figure S8D-F). These findings indicated full transcriptional activation following EZHi/DNMTi requires p300/CBP activity.

Surprisingly, blocking both H3K27ac deposition and activation of these pathways with A485, that are typically associated with therapeutic effects, did not rescue the antiproliferative effect of EZHi/DNMTi treatment and instead enhanced or instead showed anti-proliferative effects itself (Figure 6F and S8G). Our previous work demonstrated that inhibition of calcium signaling attenuated pro-inflammatory, immune, and TE responses as well in HCT116 cells, but we show here this inhibition also fails to rescue the antiproliferative effect of EZHi/DNMTi treatment (Figure S8H) (15). Notably, a transcriptional phenotype shared across EZHi/DNMTi and A485 is downregulation of oncogenic gene sets (MYC, G2M Checkpoint, E2F targets) (Figure 6D-E and S8I). These shared proliferation and transcriptional phenotypes suggested the downregulation of oncogenic pathways contributes to therapeutic efficacy in this setting devoid of an immune compartment.

Although our histone mass spectrometry showed no global loss of H3K27ac (Fig 3I), we did observe significant reductions in H3K27ac siQ-ChIP signal with EZHi treatment that primarily occurred at gene promoters and genic enhancers (Figure S6A and 6A). Significantly, we observed that H3K27ac loss at the promoters of active genes correlates with transcriptional downregulation (Figure 6G). To understand the relationship between H3K27ac loss, H3K27me1 distributions, and gene transcription, we selected gene promoters with significant H3K27ac loss within 20 kilobases of H3K27me1 signal (Figure 6H). Importantly, VD-associated H3K27ac loss was deeper, more numerous, and correlated with loss of H3K27me1 more so than TD-associated H3K27ac loss at these gene promoters (Figure 6H,I). Notably the deepest losses in H3K27me1 for both TD and VD treatment occurred across active genic regions (Figure 6I). Indeed, this epigenetic phenotype corresponds with the H3K27me1 loss observed with both TAZ and VAL in Figure 4 that occurs at active gene bodies. Next, we profiled the H3K27ac (Figure 6J), H3K27me1 (Figure 6K), and DNA methylation (Figure 6L) signal across the gene bodies. Significantly, all three epigenetic marks are reduced across these genes with EZHi/DNMTi treatment (Figure 6J-L). Consistent with this results, EZH1 has previously been shown to promote RNA polymerase II elongation for mRNA production of active genes (42,43). We observed that H3K27ac loss was deepest near and downstream of the TSS (Figure 6K), suggesting that dual EZH1/2 inhibition blocks this function of EZH1. This corresponds to transcriptional downregulation that is deepest with the VD combination (Figure 6M). However, H3K27ac and H3K27me1 loss alone is not sufficient to mediate this transcriptional downregulation as single-agent TAZ and VAL treatments induce similar loss in these epigenetic marks (Figure 6J, 6K) without transcriptional downregulation of these genes (Figure 6M).

Therefore, we hypothesized that downregulation of actively transcribed genes requires not just H3K27me1 and H3K27ac loss, but DNA hypomethylation as well. Like H3K27me1, DNA methylation levels are high across gene bodies of active genes and have been shown to regulate transcription fidelity (44). Furthermore, previous work from Yang *et al*. demonstrated that DAC-mediated DNA hypomethylation across gene bodies reduces transcriptional output, particularly of metabolic and c-MYC related pathways (45). In our treatment paradigms, DAC alone and in combination with EZHi induced differentially hypomethylated regions (DMRs) that occur throughout the genome, including genic regions of actively transcribed genes (Figure S8J). Focus on VD-associated DMRs demonstrated that highly methylated regions are most prone to DNA methylation loss, and significantly, lose H3K27me1 with TAZ and VAL treatment (Figure S8K). In support of this hypothesis, transcriptional downregulation of highly active genes is primarily observed when EZHi is combined with DNMTi (Figure 6M). Collectively, our results demonstrate that while H3K27ac deposition in VD-treated cells promotes transcriptional activation, it may not be the primary phenotype needed to mediate the therapeutic antiproliferative effect. Rather, targeting of gene body DNA methylation and H3K27me1 by DNMTi and EZHi, respectively, reduced these marks across active gene bodies resulting in attenuation of oncogenic transcriptional programs.

### EZH1 expression levels predict sensitivity to EZH2-selective inhibitors combined with DNMTi

**Figure 7.**
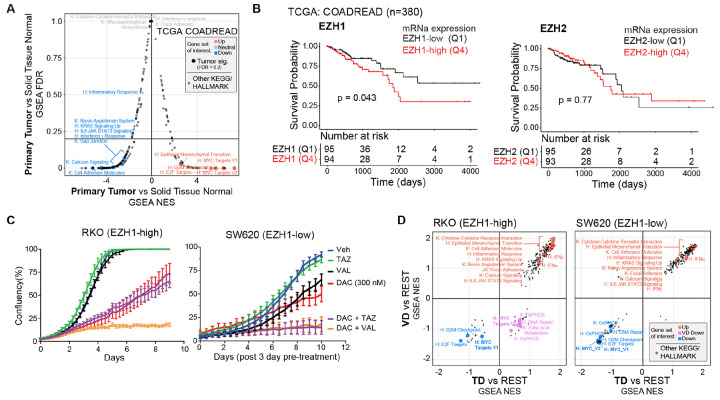
EZH1 level correlates with patient outcomes and predicts cell line sensitivity to EZH2i **A)** GSEA analysis of COADREAD patient tumors (TCGA, n = 380) versus Solid Tissue Normal. **B)** Kaplan-Meier Curves separated by EZH1 (left) and EZH2 (right) mRNA expression for the COADREAD patient cohort. The highest (Q4) and lowest (Q1) mRNA expression quartiles were used to calculate significance of overall survival. **C)** Outgrowth measurements (% confluency) of RKO or SW620 colon cancer cells treated once with DAC (300 nM) with or without valemetostat (VAL,1 μM) or tazemetostat (TAZ, 1 μM) and observed for 10 days. Data are the mean ±SEM of technical replicates from a single experiment (n=12 images per timepoint and treatment) and are representative of six (RKO) and three (SW620) biological replicates. **D)** Comparative GSEA analysis (NES) for KEGG and HALLMARK gene sets between TD (TAZ+DAC, x-axis) and VD treatment (VAL+DAC, y-axis) in RKO (left) and SW620 (right). **See also** Figure S9.

Gene set enrichment analysis (GSEA) of TCGA COADREAD patient tumors in comparison to solid tissue normal samples confirmed that pro-survival, oncogenic gene sets such as G2M checkpoint, MYC targets, and E2F targets are overexpressed in colorectal adenocarcinoma patient tumors (Figure 7A). Within this cohort, patients with the highest EZH1 mRNA expression in COADREAD tumors had overall poorer survival than patients with low EZH1 expression (Figure 7B and S9B), while EZH2 expression did not make this distinction. Given that VAL-associated treatments that dually target EZH1/2 consistently demonstrate deeper loss of H3K27me1 and H3K27ac along with transcriptional downregulation (Figure 6), we hypothesized that tumors with high EZH1 protein expression would be less sensitive to EZH2-selective inhibition when combined with DNMTi than with dual inhibition of EZH1/2. To test this hypothesis in cancer cell lines, we compared RKO, which has high expression of EZH1 at the protein level, to SW620, a colon cancer cell line expressing low levels of EZH1 (Figure 3A). Indeed, RKO cells (EZH1-high) were more sensitive to VD treatment than TD treatment while SW620 cells (EZH1-low) were equally sensitive to both VD and TD treatment (Figure 7C).

To understand the differential sensitivity of RKO cells to TD and VD treatments (and lack thereof in SW620 cells), we queried the transcriptional responses among the different drug treatments in RKO and SW620 cells. Consistent with our hypothesis, VD treatment not only had greater transcriptional activation than TD treatment but also more pronounced downregulation specific to the VD treatment (Figure S9C). In contrast, the transcriptional response in SW620 cells was similar between TD and VD treatments for both gene up- and downregulation (Figure S9C). For both RKO and SW620 cells, TD and VD treatment upregulated pro-inflammatory, immune response, and calcium signaling pathways (Figure 7D). However, as discussed in Figure 6, activation of these pathways appears to be dispensable for eliciting the antiproliferative effect of EZHi/DNMTi treatment in cancer cells. Rather, we found that VD treatment in RKO cells downregulated pro-survival, oncogenic pathways better than TD treated cells whereas TD and VD treatment of SW620 cells equally downregulated these gene sets (Figure 7D and S9D,E). Epigenetic profiling across the gene bodies of these MYC target and oxidative phosphorylation core-enrichment genes revealed that H3K27ac, H3K27me1, and DNA methylation were all significantly reduced, with VAL-associated treatments demonstrating the deepest loss in H3K27ac and H3K27me1 in RKO (Figure S9F). We validated these findings at the protein level in HCT116 cells, which we previously reported also respond to combined EZHi/DNMTi treatment (Figure 2) (15), that, indeed, combination EZHi/DNMTi treatment results in both c-MYC and MYC target protein reduction (Figure S9G).

## Discussion

Our group previously demonstrated enhanced efficacy in activating anti-tumor transcriptional programs when combining DNMTi with EZH2-specific inhibition (15). In this study, we expanded on this previous research to show that using a dual EZH1/2 inhibitor in combination with DNMTi shows superior efficacy at rewiring transcriptional pathways and reducing cell proliferation, particularly in cell lines less sensitive to EZH2 specific inhibition in combination with DNMTi.

EZH1 and EZH2 are mutually exclusive components of the PRC2 complex. EZH2 has been well-characterized as the main catalytic subunit of the PRC2 complex, while the contributions of EZH1 to PRC2-mediated activity remain less understood. Our study supports EZH2 as the main driver of H3K27me2 and H3K27me3 deposition, but here we further show that EZH1 is the main contributor to the H3K27 mono-methylation mark in the absence of EZH2. We hypothesized that H3K27me1 has an important regulatory role in the absence of EZH2 and higher order methylation. Indeed, when we inhibit EZH2 pharmacologically or genetically, we observe a notable increase in the H2K27me1 despite a significant decrease in the di- and tri- methylation states. Mechanistically, this suggests EZH2-containing PRC2 has a preference for depositing H3K27me2/3, and therefore, H3K27me1 may accumulate as a result of deficient catalysis to di- and trimethylation. Alternatively, EZH1-containing PRC2 may deposit more H3K27me1 to compensate for the loss of me2/me3 in transcriptionally repressed regions of the genome. Our results suggest perhaps both mechanisms are at play as the resultant H3K27me1 maintenance we observed primarily occurred at genomic regions associated with developmental and cell identity processes marked by deep Polycomb repression.

While previous literature has mainly focused on H3K27me1 as a gene body mark associated with active gene expression, analogous to DNA methylation across gene bodies (33), here we describe a previously unrecognized regulatory role in which EZH1-mediated H3K27me1 blocks the redistribution of H3K27ac activating marks. We show that combination treatment with DNMTi and dual EZH1/2i inhibition leads to loss of all three methylation states of H3K27, a depletion of DNA methylation, and redistribution of activating H3K27ac marks towards bivalent regions of genome. The regions that gain H3K27ac are dependent on p300/CBP activity for transcriptional activation and enriched for anti-tumor gene profiles such as viral mimicry, calcium signaling, and immune response. Unexpectedly, we also observed redistribution of H3K27ac away from genes driving oncogenic transcription programs, including MYC and MYC target genes. We hypothesize that redistribution of H3K27ac away from these genes likely contributes to the therapeutic effectiveness of the dual inhibitor in addition to gene activation.

Finally, we identify a combinatorial epigenetic mark where certain regions of the genome, particularly transcriptionally active gene bodies, are dually marked by DNA methylation and baseline H3K27 monomethylation. Combination treatment with either PRC2i and DNMTi depletes both of these PTMs. When we treat cells with the EZH2-selective TAZ, we observe a gain in H3K27me1, specifically in transcriptionally repressed regions previously marked by H3K27me3 and DNA methylation. When we treat with VAL, we eliminate this gain in the H3K27me1 mark and see greater redistribution of the H3K27ac mark. These results suggest a direct interplay between these PTMs regulating transcriptional changes induced by these drugs. Furthermore, these results suggest that therapeutic efficacy of EZHi/DNMTi combination treatment is mediated through transcriptional downregulation by DNMTi-associated gene body hypomethylation and EZHi-associated H3K27me1 reduction across gene bodies of oncogenic transcriptional programs.

As the clinical testing of EZH2-specific inhibitors expands in popularity, our work indicates the potential benefit of extending this to dual EZH1/2i (46). There is current FDA approval for TAZ in epithelioid sarcoma, which exhibits PRC2 hyperactivation resulting from SWI/SNF complex mutations, and follicular lymphoma, characterized by EZH2 gain-of-function mutations (8,9). SWI/SNF and PRC2 are opposing, major regulators of chromatin structure and gene expression. Many solid tumors have mutations in SWI/SNF that cause PRC2 hyperactivity; for example, colon cancer cells can have ARID1A and SMARCA4 driver mutations (47). Additionally, targeting EZH1 with dual EZH1/2i in slow-growing or non-proliferative tumors may be beneficial as studies suggest quiescent cells primarily utilize EZH1-containing PRC2 (48). Colon cancer cells treated with DNA hypomethylating agents also have higher PRC2 activity and thus more H3K27me3, which provides further rationale for targeting PRC2 in combination with DNA hypomethylating therapy. These examples highlight important new potential uses for these drugs apart from their current FDA approved usage.

Most trials with epigenetic therapies in solid tumors have been conducted in heavily pre-treated populations with severely disrupted epigenomes; this has limited the available information on the patients most likely to respond to these therapies. Additionally, these populations do not exhibit PRC2 hyperactivity until DNMTi therapy as noted above. Therefore, PRC2 hyperactivity itself is not predictive of sensitivity to combined EZHi/DNMTi. This study provides rationale for using EZH1 as a biomarker in patient populations to determine individuals most likely to respond to TAZ/DNMTi and further indicates the preferential use of VAL/DNMTi for patients with high EZH1. Future directions may include identifying concurrent biomarkers that would aid clinical decisions for using a single vs dual EZHi inhibitor.

## Methods

### Cell culture

HCT116 (ATCC CCL-247), RKO (ATCC CRL-2577), SW620, (ATCC CCL-227) colon cancer cell lines and HEK293T (ATCC CRL-3216) were purchased from American Type Culture Collection (ATCC) and maintained according to ATCC recommendations. HCT116 (wild-type and the NLuc reporter line), SW620, and HEK293T were maintained in McCoy’s (Gibco 16600-082), Leibovitz's L-15 (ATCC 30-2008), or DMEM (Gibco 11995-065) culture media, respectively. RKO were maintained in RPMI 1640 (Gibco 11875-093) culture medium supplemented with L-glutamine (Gibco 25030-081), 0.1 mM non-essential amino acids (Gibco 11140-050), 1 mM Sodium Pyruvate (Gibco 11360070). All media were supplemented with 10% Fetal Bovine Serum (FBS, Millipore Sigma F0926) and 1% penicillin/streptomycin (Life Technologies 15140-122) at 5% CO_2_ and 37°C.

### CRISPR/Cas9-mediated knockout cell line generation

Cloning was performed as previously reported (49). Briefly, pSpCas9(BB)-2A-Puro (PX459) V2.0 (Addgene plasmid #62988) was digested with restriction enzyme BbsI-HF (New England Biolabs R3539L) in rCUTSMART buffer (New England Biolabs B6004S) and purified using Monarch Spin DNA Gel Extraction and Monarch Spin PCR & DNA Cleanup Kits (New England Biolabs T1120S and T1130S). Oligo pairs for genes (see below) of interest were annealed and ligated into the digested vector using T4 ligase (ThermoFisher EL0014) then transformed into XL-10 Gold ultracompetent bacterial cells (Agilent 200315). Miniprepped plasmids (Qiagen 27104) were confirmed by whole plasmid sequencing through Plasmidsaurus.

**Table:** 

Loci	Forward gRNA (5’-3’)	Reverse gRNA (5’-3’)
*EZH1*	CACCGAGACTAAAGAAGGAGACAGTGACA	AAACTGTCACTGTCTCCTTCTTTAGTCTC
*EZH2*	CACCGATCTATGTTGGGGGTACATTCAGG	AAACCCTGAATGTACCCCCAACATAGAT
Chr12 Gene Desert, (HG38, chr12:127711096-127713493)	CACCGTATTCCAACGGTGACTCAACCCAG	AAACCTGGGTTGAGTCACCGTTGGAATAC

Plasmids were transfected into target cell lines at a 1 μg:2 μL ratio with XtremeGene HP (Sigma 6366244001) and selected with 2 μg/mL Puromycin (Sigma P8833) for 48 hr. Cells were split into single cell clonal populations through limiting dilution and individual knock out clones were confirmed by western blotting for the protein of interest and Sanger sequencing of the CRISPR guide region. To analyze mutations or indels on each allele for an individual clone, we first amplified a ~400bp region around the CRISPR guide cut site (EZH2 For 5’-ACCATGCACAATATTTAGTTGGCT, EZH2 Rev 5’-TGATAGCACTCTCCAAGCTGC-3', EZH1 For 5’-GTTTTGTTAAGCAGCTCTCATTGT-3', EZH1 Rev 5’-CAGTGATCCTGGGTTTACCAGAG-3'), ligated the amplicon into a pGEM-T vector (Promega A1360), and transformed the plasmid into JM109 cells (Promega L2001) according to manufacturer’s guidelines. Plasmid DNA was amplified directly from bacterial colonies in 96 well plates through rolling PCR amplification using Temphliphi according to manufacturer’s protocol (GE Healthcare 25-6400-10) and sent to Genewiz for Sanger sequencing.

### Inducible shRNA cell line generation

The shRNA cell lines were generated as outlined in the protocol provided by AddGene for the pLKO1 system for cloning and inducible shRNA expression (AddGene 21915) (50,51). Briefly, the TET-pLKO-puro plasmid (Addgene 21915) was digested with AgeI and EcoRI, and the fragment was gel purified. shRNA oligos were selected from the Broad Institute RNAi consortium shRNA library list. Oligos were ordered, annealed, and then ligated into the digested plasmid. Plasmid sequence was confirmed through Plasmidsaurus.

Plasmid for the doxycycline-inducible shEZH1 was transfected into HEK293T (ATCC CRL-3216) cells with pPAX2 (Addgene 12260) and pMD2.G (Addgene 12259) packaging plasmids to make lentivirus. A RKO Chr12 CRISPR control clone (clone B2) or EZH2 CRISPR KO clone (clone A8-5) were infected with virus media mixed with 1 mg/mL polybrene (Millipore TR-1003-G) followed by selection with 2 μg/mL puromycin (Sigma P8833). To test knockdown, cells were treated with 20 ng/mL doxycycline (Cayman Chemical 14422) for 72 hr, collected, Western blotted as below, and probed with an EZH1 antibody (Cell Signaling 42088).

### Small molecule preparation and use

5-aza-2’-deoxycytidine (DAC or decitabine, Sigma A3656), A485 (Cayman Chemicals 24119), A-395 (Cayman Chemicals 20257), CPI-169 (Selleck Chemicals S7616), CPI-1205 (Selleck Chemicals S8353), EED226 (MedChemExpress HY-101117), Cyclosporin A (CsA, Selleck Chemicals S2286), EEDi-5285 (MedChemExpress HY-136977), EPZ0011989 (Selleck Chemicals S7805), EPZ005687 (Cayman Chemical 13966), EZH2 Degrader-2 (MedChemExpress HY-157164), GSK126 (Selleck Chemicals S7061), GSK343 (Selleck Chemicals S7164), GSK503 (Cayman Chemical 18531), MAK683 (MedChemExpress S8983), mevrometostat (MEV or PF-1497, Chemietek CT-PF0682), MS177 (Selleck Chemicals E1163), MS1943 (MedChemExpress HY-133129), ORIC-944 (Selleck Chemicals E1967), tazemetostat (TAZ or EPZ6438, Selleck Chemicals S7128), tulmimetostat (TUL or CPI-0209, Selleck Chemicals E1497), UNC1999 (Caymen Chemical 14621), valemetostat (VAL or DS-3210b, Chemietek CT-DS3201), and UNC6852 (MedChemExpress HY-130708) were dissolved in 100% dimethyl sulfoxide (DMSO) at 5 or 10 μM and stored at −20°C. Attached cells were treated with each drug or vehicle (DMSO, % equivalent) for 72 hours with no media change.

### Cell viability and outgrowth assays

To measure cell viability, cells were plated in 96-well plates at a density of 3-4000 cells/well and treated the following day. The CellTiter-Fluor (Promega G6080) kit was used according to the manufacturer’s protocol, and the fluorescence signal was measured using a BiotTek SynergyNeo2 Microplate Reader. Background signal from media-only wells was subtracted to obtain the final reported relative fluorescence units (RFU).

To measure RKO and SW620 cell outgrowth over time, ~5000 cells/well were plated into 24-well dishes and allowed to adhere overnight. The following day, epigenetic drugs including DAC (300 nM) with or without TAZ or VAL (1 μM) and/or A485 (10 μM) in fresh media were applied to the adhered cells. For each well and treatment, a Sartorius Incucyte S3 captured 9-12 brightfield images at each timepoint, and the average confluency (%) per well and timepoint was calculated using the Sartorius software.

To measure outgrowth of HCT116 following pretreatment, 100,000 cells/well were plated in a six-well dish and adhered cells were treated with epigenetic drugs (DAC [30 nM] with or without TAZ or VAL [1 μM]) and/or CsA (5 μM) in fresh media the following day. After 72 hrs, cells were replated and retreated for a second 72 hr treatment cycles for a total of 6 days pretreatment. At 6 days, 40,000 cells/well were plated into a six-well dish and re-treated with drugs for a third time, which is represented as “day 0” following pretreatments on confluency graphs. The Sartorius Incucyte S3 captured 12 brightfield images at each timepoint, and the average confluency (%) per well and timepoint was calculated using the Sartorius software.

### *SFRP1*-NanoLuciferase cell reporter assay

We previously reported on engineering the *SFRP1*-Nanoluciferase cell reporter cell line (15). Briefly, a NanoLuciferase (NLuc) cassette was previously inserted into exon 2 of an endogenous *SFRP1* allele in DNMT1-hypomorph HCT116 colon cancer cells with reduced DNA methylation (19). To measure NLuc activity in *SFRP1*-NLuc reporter cells, the CellTiter-Fluor assay was duplexed with the Nano-Glo Luciferase Assay System (Promega N1110). First, cells were plated and treated in 96-well plates as above for the cell viability assays. To duplex the assays, CellTiter-Fluor was used at a 5x concentration preceding the Nano-Glo assay. NanoGlo reagent was applied according to the manufacturer’s protocol, and a BioTek SynergyNeo2 Microplate Reader was used to obtain relative luminescence units (RLU). After background subtraction from media-only wells, RLU were normalized to CellTiter-Fluor RFU to account for cell viability.

### siRNA transfection

ON-TARGETplus siRNA SMARTpools (5 μl of 20 μM stock) targeting DNMT1, EZH1, EZH2, MYC, or SUZ12 (Dharmacon L-004605-00-0005, L-004217-00-0005, L-004218-00-0005, L-003282-02-0005, and L-006957-00-0010) and non-targeting control siRNA pools (Dharmacon D-001810-10-05) or Lipofectamine RNAiMax (5 μL, ThermoFisher 13778075) were each separately diluted in 100 uL Optimem (Life Technologies 31985-062). Diluted siRNA and RNAiMax were mixed together 1:1, and incubated for 20 min. McCoy’s Media, with or without DAC (30 nM) and supplemented with 10% FBS but no antibiotics, was refreshed on attached cells that had been plated the previous day at 100,000/well in a 6-well dish. The siRNA/Lipid mixture was added dropwise to the fresh media for a final siRNA concentration of 50 nM. Cells were collected 72 hrs after transfection or split and collected 96 hours later (seven days total).

### Genome-wide siRNA screen

The NLuc reporter cell line was used in conjunction with a genome-wide siRNA library (Human ON-TARGETplus siRNA Library – Whole Genome – SMARTpool, Dharmacon G-105005). Library siRNA pools contained in 384 well plates were resuspended in 1x siRNA buffer (Dharmacon B-002000-UB-100) at 2 μM for long term storage at −80°C and intermediate daughter plates were diluted to 500 nM in RNase-free H_2_O for short term storage at −20°C. To reverse-transfect cells, 1 μl of diluted Oligofectamine transfection reagent (ThermoFisher 1225201, 0.09 μL in 0.91 μL RNase-free H_2_O) was added to each well using a Viaflo384 electronic pipette liquid handler (Integra Biosciences). Next, 4.5 μL of each siRNA pool from the daughter plates was added to individual wells. Plates were centrifuged at 300g for 1 min to mix Oiligofectamine and siRNA and were then incubated for 20-30 min. During this incubation, NLuc reporter cells were trypsinized, counted, and resuspended in McCoy’s medium supplemented with 10% FBS without antibiotics; ~275 cells in 39.5 μL media were added to each well, bringing the final siRNA concentration to 50 nM. Plates were again centrifuged briefly at 300 g and incubated at 5% CO_2_ and 37°C. Given the size of the screen and capacity of the Viaflo Liquid Handler, the screen was split into four parts, and all components and steps maintained exactly the same.

After seven days, a Sartorius Incucyte S3 was used to capture brightfield images of each well to capture confluency (%) as a proxy for cell number and viability (Figure S1A). 45 μL of NanoGlo reagent was applied according to the manufacturer’s protocol, and a BioTek SynergyNeo2 Microplate Reader was used to obtain relative luminescence units (RLU). After background subtraction from media-only wells, RLU were normalized to confluency (%) to account for cell number. To exclude false positives, we omitted genes that are very lowly or not expressed (CPM<100) in wild-type HCT116 basal state or when treated with a low dose of DAC (to reflect the DNMT1-hypomorph status in the NLuc reporter) using previously reported HCT116 RNA-seq data (15). To exclude genes that induce cell death, which we have not reported as a phenotype directly associated with *SFRP1* reactivation in these cells, we omitted genes whose knockdown decreased confluency by more than one standard deviation from the mean (Figure S1B). Knockdown of WRN, an HCT116 essential gene according to DEPMAP CRISPR and RNAi screens (https://depmap.org/portal) (52), acted as a positive control for transfection efficiency (Figure S1C), and NLuc activity following DNMT1 knockdown served as a positive control for *SFRP1* reactivation based on our previously reported findings (Figure 1C) (15).

### RNA isolation, cDNA synthesis, and qRT-PCR

TRIzol Reagent (Invitrogen 15596026) was added directly to adhered and treated cells, collected, and stored at −80°C. After thawing, total RNA was extracted following the manufacturer’s protocol, and RNA was resuspended in DEPC Nuclease-Free water. cDNA was synthesized from 2 μg RNA using a High-Capacity cDNA Reverse Transcription Kit (Applied Biosystems 4368814) according to the manufacturer’s protocol with the addition of RNAse inhibitor (Life Technologies N8080119). Using the KAPA SYBR FAST qPCR Kit (Roche 07959567001) according to the manufacturer’s protocol, qRT-PCR analyzing technical duplicates for each gene (see below) and sample was performed on a BioRAD CFX Opus93 Real-Time PCR System. Data was analyzed using 2^−dd(Ct)^ method (53) where the fold change was determined by normalizing to the *RPL4* housekeeping gene and a control (siNTC) sample.

**Table:** 

Gene	Forward (5’-3’)	Reverse (5’-3’)
*IFI27*	TCTGGCTCTGCCGTAGTTTT	GAACTTGGTCAATCCGGAGA
*RPL4*	ATCCAAAGAGCCCTTCGAGC	CTGGCGAAGAATGGTGTTCC
*SFRP1*	CATGCAGTTCTTCGGCTTCT	GATTTCAACTCGTTGTCACAGG

### Western blotting

Cells were lysed on ice in cold CSK lysis buffer as previously reported (15). 2-5 μg of total protein to probe for histones or 10-15 μg of total protein for other targets were size-separated by SDS-PAGE and transferred to a PVDF membrane. Membranes were blocked for one hour at room temp (PBS, 0.1% Tween-20, and 5% BSA), washed in PBST (PBS and 0.1% Tween-20), and incubated in blocking buffer overnight at 4°C with the following antibodies: β-Actin (1:1,000; Cell Signaling Technologies 4970), β-Tubulin (1:50,000; Protein Tech 66240), DNMT1 (1:1,000; Abcam ab134148), EED (1:1,000; Cell Signaling Technology 85322), EZH1 (1:1,000; Cell Signaling Technology 42088), EZH2 (1:1,000; Cell Signaling Technology 5246), H3 (1:50,000; EpiCypher 13-0001), H3K18ac (1:1,000; Invitrogen MA5-24669), H3K27ac (1:1,000; Active Motif 39133), H3K27me1 (1:1,000; Active Motif 61015), H3K27me2 (1:2,000; Cell Signaling Technology 9728), H3K27me3 (1:2,000; Cell Signaling Technology 9733), and SUZ12 (1:1,000; Abcam 12073). Histone antibodies were chosen for their high specificity and selectivity among antibodies profiled by our lab on histone peptide arrays (histoneantibodies.com) (54). The following day, membranes were washed in PBST and incubated in HRP-conjugated secondary antibody (1:10,000; Sigma-Aldrich GENA934) for 1 hour at room temp, washed again in PBST, incubated in Enhanced Chemiluminescence substrate, and imaged with film.

### Histone acid extraction and histone PTM mass spectrometry

For analysis of histone PTMs by mass spectrometry, histones were isolated as previously described (55). Pellets from RKO cells treated with epigenetic drugs or with CRISPR mutations were lysed in hypotonic lysis buffer (10 mM Tris–HCl pH 8.0, 1 mM KCl, 1.5 mM MgCl_2_ supplemented with 1 mM DTT, 1 mM PMSF, 5 mM sodium butyrate, EDTA-free Roche mini protease inhibitor tablet, and phosphatase inhibitor tablet) while rotating at 4°C for 30 min. Intact nuclei were then pelleted by centrifugation at 10,000xg for 10 min at 4°C. Nuclei were then incubated in cold 0.2 M H_2_SO_4_ for 2-4 hr rotating at 4°C, and centrifuged at 16,000xg for 10 min at 4°C. Next, histones were precipitated by adding cold TCA to 33% v/v dropwise to the supernatant and incubated 1 hr up to overnight while rotating at 4°C. Precipitated histones were centrifuged at 16,000xg for 10 min at 4°C, washed with cold acetone/0.1% HCl, pelleted at 3,400xg for 5 min at 4°C, and washed in cold acetone alone. A glass Pasteur pipette was used to gently wash the histones down the sides of the tubes during the wash steps. Samples were airdried for 20 min on the benchtop and then submitted as dried histones or resuspended in water. Finally, histone extracts were digested and processed as previously described in the Sidoli lab (16,56) or by in the VAI Mass Spectrometry Core as detailed below.

### Sample preparation for histone proteomics by VAI Mass Spectrometry Core

Purified histones in 100 mM ammonium bicarbonate (Sigma, 09830) with 20% acetonitrile (Fisher Chemical, LC/MS grade), were propionylated with 5 μL propionic anhydride (Sigma, 240311) immediately followed by the addition of 10 μL ammonium hydroxide (Fisher, A470) and adjusted to a pH ~8 and incubated for 10 minutes at room temperature. Propionylation was repeated prior to overnight tryptic digestion (Promega, V5073). After digestion, samples were propionylated twice, as before, and dried in a speedvac concentrator. The digest was cleaned using an Ultra-Micro C18 Spin Column (Harvard Apparatus, 74-7206), dried, and resuspended at 1μg/μL in 0.1% trifluoroacetic acid (Sigma, T6508) for mass spectrometry analysis.

### Data-independent acquisition (DIA) LC-MS/MS proteomics for histones

DIA analyses were performed on Orbitrap Exploris 480 coupled to Vanquish Neo system (Thermo Fisher Scientific). 1 μg of digested peptides were separated on a nano capillary column (20 cm × 75 μm I.D., 365 μm O.D., 1.7 μm C18, CoAnn Technologies, Washington, # HEB07502001718IWF) at 300 nL/min. Mobile phase A consisted of LC/MS grade H_2_O (LS118- 500, Fisher Scientific), mobile phase B consisted of 20% LC/MS grade and H_2_O and 80% LC/MS grade acetonitrile (LS122500, Fisher Scientific), both containing 0.1% FA. The LC gradient was: 3% B to 30% B in 51 min, 80% B in 5 min, 98% B in 2 minutes and held 98% B for 3 min, with a total gradient length of 60 min. The column temperature was kept constant at 45 °C using a customized column heater (Phoenix S&T, Chadds Ford, PA). Full MS spectra were collected at 120,000 resolution (full width half-maximum; FWHM), and MS2 spectra at 30,000 resolution (FMWH). Both the standard automatic gain control (AGC) target and the automatic maximum injection time were selected. A precursor range of 300-1000 m/z was set for MS2 scans, and an isolation window of 30 m/z was chosen with a 1 m/z overlap for each scan cycle. 32% HCD collision energy was used for MS2 fragmentation. DIA data was processed in EpiProfile (version 2.1) (57). The manufacturer’s default parameters were used.

### Sample Preparation for Untargeted Global Proteomics

Samples were processed using the EasyPep Maxi MS Sample Prep Kit (A40006, Thermo Fisher Scientific) according to manufacturer instructions. Briefly, cells were resuspended in 50 μL of lysis solution and proteins were quantified using the Pierce BCA Protein Assay Kit (23227, Thermo Fisher Scientific) following the vendor supplied protocol. Plates were read at an absorbance of 562 nm using the Synergy LX Multi-Mode Reader and Gen5 software was used for data analysis (BioTek/Agilent). Polynomial regression was used in the Gen5 software to calculate protein concentrations to a protein standard curve after an average blank absorbance subtraction. 25 μg of protein per sample was reduced and alkylated at 95°C for 10 min, and samples were digested overnight with Trypsin/Lys-C at 30°C at a ratio of 10:1 (protein:enzyme (w/w)). Resulting peptides were cleaned with EasyPep maxi kit supplied peptide clean up columns and dried down in a Genevac SpeedVac prior to resuspension for instrument analysis. Samples were resuspended in 12.5 μL 0.1% formic acid (FA) (LS118-1, Fisher Scientific) and diluted 1:1 with 6 μL of 0.1% trifluoroacetic acid (TFA) (LS119-500, Fisher Scientific) in autosampler vials.

### Data-independent acquisition (DIA) LC-MS/MS for global proteomics

DIA analyses were performed on Orbitrap Eclipse coupled to Vanquish Neo system (Thermo Fisher Scientific) with a FAIMS Pro source (Thermo Fisher Scientific) located between the nanoESI source and the mass spectrometer. 2 μg of digested peptides were separated on a nano capillary column (20 cm × 75 μm I.D., 365 μm O.D., 1.7 μm C18, CoAnn Technologies, Washington, # HEB07502001718IWF) at 300 nL/min. Mobile phase A consisted of LC/MS grade H_2_O (LS118- 500, Fisher Scientific), mobile phase B consisted of 20% LC/MS grade and H_2_O and 80% LC/MS grade acetonitrile (LS122500, Fisher Scientific), both containing 0.1% FA. The LC gradient was: 1% B to 24% B in 110 min, 85% B in 5 min, and 98% B for 5 min, with a total gradient length of 120 min. The column temperature was kept constant at 50 °C using a customized column heater (Phoenix S&T, Chadds Ford, PA). For FAIMS, the selected compensation voltage (CV) was applied (−40V, −55V, and −70V) throughout the LC-MS/MS runs. Full MS spectra were collected at 120,000 resolution (full width half-maximum; FWHM), and MS2 spectra at 30,000 resolution (FMWH). Both the standard automatic gain control (AGC) target and the automatic maximum injection time were selected. A precursor range of 380-985 m/z was set for MS2 scans, and an isolation window of 50 m/z was chosen with a 1 m/z overlap for each scan cycle. 32% HCD collision energy was used for MS2 fragmentation.

DIA data was processed in Spectronaut (version 18, Biognosys, Switzerland) using direct DIA. Data was searched against the *Homo sapiens* proteome. The manufacturer’s default parameters were used. Briefly, trypsin/P was set as digestion enzyme and two missed cleavages were allowed. Cysteine carbamidomethylation was set as fixed modification, and methionine oxidation and protein N-terminus acetylation as variable modifications. Identification was performed using a 1% q-value cutoff on precursor and protein levels. Both peptide precursors and protein false discovery rate (FDR) were controlled at 1%. Ion chromatograms of fragment ions were used for quantification. For each targeted ion, the area under the curve between the XIC peak boundaries was calculated.

### Antibody validation for chromatin immunoprecipitation

Antibodies were first analyzed for binding specificity using the histone peptide array as previously described by our lab (54,58). Briefly, we incubated candidate antibodies with slides previously printed with a known library of histone peptides of various modifications and blocked in hybridization buffer (1X PBS pH 7.6, 0.1% Tween, 5% BSA). Slides were washed and then incubated with Alexa Fluor 647 conjugated secondary antibody (1:5000, Life technologies A21245) before scanning with an Innopsys scanner. Slides were analyzed and peptide binding was determined using ArrayNinja (59).

For antibody titrations, a fixed amount of chromatin (5 μg) was incubated with increasing amounts of antibody. Total DNA mass immunoprecipitated was measured by Qubit dsDNA High Sensitivity kit (Invitrogen Q32854).

### Chromatin Immunoprecipitation (ChIP)

Cells exposed to drugs for 72 hours were fixed in buffer (1% formaldehyde, 1X DPBS) for 10 min at room temp with shaking, quenched with 125 mM glycine 5 min at room temp, scraped into cold DPBS, washed 2X with cold DPBS, flash frozen in liquid N2, and stored at −80°C until use. Thawed pellets were lysed in 1 mL Cell Lysis Buffer (5 mM PIPES ph8.0, 85 mM KCl, 1% NP40, 100 mM PMSF, Roche EDTA-free protease inhibitor cocktail) for 20 min with rotation at 4°C and cleared by centrifugation at 2000 x g for 5 min at 4°C. Pelleted nuclei were washed in cold Micrococcal nuclease (Mnase) digestion buffer (50 mM Tris-HCl pH 8.0, 1mM CaCl_2_, 300mM sucrose, 100 mM PMSF, Roche EDTA-free protease inhibitor cocktail), pelleted by centrifuge, resuspended in 1 mL 37°C Mnase buffer with 50U Mnase (Worthington LS004798), and incubated at 37°C shaking vigorously for 12 min. Digestion was stopped by addition of 10 mM EDTA and nuclei were transferred to a 1 mL milliTUBE (Covaris). Chromatin was sheared to a range of 300-600 base-pair fragments using a Covaris E220 evolution Focused ultrasonicator with the following parameters: Peak power (140.0), Duty Factor (5.0), Cycles/Burst (200), Duration (120 seconds), Temperature (4°C). Samples were centrifuged at 13,000 rpm at 4°C for 10 min and supernatants collected and stored at −80°C.

To quantify chromatin, 20 μL was sampled from the 1 mL prepped chromatin and added to 80 μL elution buffer (50 mM NaHCO_3_, 1% SDS), 0.3 M NaCl, and 200 μg/mL proteinase K. Samples were incubated at 67°C overnight to reverse crosslink and remove protein, which was followed by incubation with RNase A/T (Invitrogen EN0551) at 37°C for 30 min to remove RNA. DNA was purified using the Qiagen PCR purification kit (Qiagen 28104) and quantified by Qubit. Correct DNA fragment size was confirmed by running on a 2% agarose gel.

For immunoprecipitation (IP), 5 μg of prepared chromatin was incubated with either 2.5 μL (2.5 μg) of H3K27ac antibody (ActiveMotif 39133, lot 31521015) or 5 μL of H3K27me1 antibody (ActiveMotif 61015, lot 03822017) overnight at 4°C with constant rotation. 5% input of normalized chromatin was removed and set aside. The next day, 30 μL of Dynabeads Protein G magnetic beads (ThermoFisher 10004D) was added and samples were incubated for 3 hours at 4°C with rotation. Bead-immuno-chromatin complexes were then washed 3X for 5 min with rotation at room temperature with low salt WB1 (20 mM Tris-HCl pH 8.0, 150 mM NaCl, 2 mM EDTA, 1% Triton X-100, 0.1% SDS), 3X with high salt WB2 (20 mM Tris-HCl pH 8.0, 500 mM NaCl, 2 mM EDTA, 1% Triton X-100, 0.1% SDS), 1X with WB3 (20 mM Tris-HCl pH 8.0, 250 mM LiCl, 1 mM EDTA, 1% NP-40, 1% Na-deoxycholate) and 1X with TE (10 mM Tris-HCl pH 8.0, 1 mM EDTA pH 8.0). Beads were incubated in 100 μl of Elution Buffer shaking at 65°C for 30 min to elute immuno-chromatin complexes. Reverse crosslinking, protein digestion, RNA digestion, and DNA clean up were performed as above for both the IPs and the input samples to both quantify and isolate DNA for sequencing.

### siQ-ChIP library preparation and sequencing

Immunoprecipitated fragments and saved inputs were quantified with a Qubit dsDNA High Sensitivity Assay kit (Invitrogen Q32851), and 10 ng of purified DNA for each IP and input sample were used for library preparation with the KAPA Hyper Prep Kit (Kapa Biosystems KR0961). Library preparation including fragment end-repair, A-tail extension, and adapter ligation was conducted per the manufacturer’s instructions (KAPA). Adapter-ligated fragments were amplified with 11 cycles following the recommended thermocycler program, and DNA was purified with two rounds of purification using KAPA Pure Beads (KK8000). Quality and quantity of the finished libraries were assessed using a combination of Agilent DNA High Sensitivity chip (Agilent Technologies, Inc.), QuantiFluor^®^ dsDNA System (Promega Corp., Madison, WI, USA), and Kapa Illumina Library Quantification qPCR assays (Kapa Biosystems). Individually indexed libraries were pooled and 50 bp, paired end sequencing was performed on an Illumina NovaSeq6000 sequencer using an S2, 100 bp sequencing kit to a minimum read depth of 50M read pairs per IP library and 100M read pairs per Input library. Base calling was done by Illumina RTA3 and output of NCS was demultiplexed and converted to FastQ format with Illumina Bcl2fastq (v1.9.0).

### siQ-ChIP-seq processing and analysis

siQ-ChIP sequencing reads were 3’ trimmed and filtered for quality and adapter content using TrimGalore (v0.5.0) and quality was assessed by FastQC (v0.11.8). Reads were aligned to human assembly hg38 with bowtie2 (v2.3.5, RRID:SCR_016368) and were deduplicated using removeDups from samblaster (v.0.1.24) (60). Aligned BAM files were used for quality control analysis with “deeptools” (v3.2.0) ‘plotFingerprint’ and ‘plotPCA’ functions. Aligned SAM files were then processed for pair-end reads with high mapping quality (MAQ ≥ 20), correct pair orientation (Sam Flags = 99, 163), and fragment length as described for siQ-ChIP (https://github.com/BradleyDickson/siQ-ChIP). Param.in files were prepared for each sample with all required parameters and measurements required for siQ-ChIP normalization. IP tracks (with siQ-ChIP efficiency values) and comparative responses between drug treatments (relative to VEH) were generated with execution of getsiq.sh (version: September 2024) with the EXPlayout file (NOTE: params.in and EXPlayout file will be provided with the GEO accession). Each individual inhibitor-treated biological replicate was compared to each individual vehicle-treated biological replicate.

To determine the change in H3K27ac distributions between inhibitor-treated samples and vehicle-treated samples, each inhibitor-treated biological replicate (e.g. DAC1) was individually compared to VEH-treated biological replicates (e.g. DAC1vsVeh1, DAC1vsVeh2), and the average log_2_ fold-change in response (area of peak in inhibitor-treatment/area of peak in vehicle treatment) was calculated. Peaks were considered conserved among biological replicates using GenomicRanges (v1.56.2) ‘findOverlaps’ function. Finally, the average log_2_ fold-change in response was calculated for the peaks conserved between the two inhibitor-treated biological replicates. For H3K27ac, peaks were considered significant if the log_2_ fold-change in response was ≥ 0.585 (increase) or ≤ −1.0 (decrease). For H3K27me1, the variance (in general high among the 3 biological replicates) (≤ 1) was considered for significance peak calling along with log_2_ fold-change ≤ −0.5 (decrease) or ≥ 0.5 (increase). Gaussian Mixture Modeling was used to separate H3K27me1 peaks into ‘narrow’ and ‘broad’ categories using the R package ‘mcluster’ (v6.1.1) (61).

### ChromHMM (v1.23)

Enrichment overlap analysis with siQ-ChIP peaks genes were conducted with the ‘OverlapEnrichment’ function using the genomic coordinates for each dataset and the publicly available 18-state chromHMM annotation for parental RKO cells (ENCODE ENCSR974TXE) (62). Enrichment bias for chromHMM states took the percentage of total enrichment within a sample. 18-state chromHMM annotations were condensed into general genomic annotation categories as follows: Promoter (TssA, TssFlnk, TssFlnkD, TssFlnkU, TssBiv), Enhancer (EnhBiv, EnhA1, EnhA2, EnhWk, EnhG1, EnhG2), Genic (TxWk, Tx), and Repressive (ReprPCWk, ReprPC, Het, Quies, ZNF/Rpts).

### Genomic DNA isolation

Genomic DNA was extracted using the DNeasy Blood & Tissue Kit (Qiagen 69504) following the standard protocol. Samples were then treated with 1 mg/ml RNAse A at 37°C for 30 minutes. DNA was re-precipitated with 1/10 volume 3 M sodium acetate pH 4.8 and 2.5 volumes 100% ethanol and stored overnight at −20°C. Precipitated DNA was pelleted by centrifugation at 17,090 x g for 30 minutes at 4°C. The pelleted DNA was washed twice with 70% ethanol, allowed to dry for 15 minutes, and resuspended in nuclease-free water.

### Infinium MethylationEPIC BeadChip (EPIC array)

Genomic DNA was quantified with the Qubit dsDNA High Sensitivity Assay kit (Invitrogen Q32851), and 1.5 μg of genomic DNA was submitted to the VAI Genomics Core (RRID:SCR_022913) for quality control analysis, bisulfite conversion, and DNA methylation quantification using the Infinium MethylationEPIC BeadChIP (Illumina) processed on an Illumina iScan system following the manufacturer’s standard protocol (63,64).

### EPIC array data processing and analysis

All analyses were conducted in the R statistical software (v4.4.1) (R Core Team). Raw IDAT files for each sample were processed using the Bioconductor (RRID:SCR_006442) package “SeSAMe” (version 1.22.2) for extraction of probe signal intensity values, normalization of probe signal intensity values, and calculation of β-values from the normalized probe signal intensity values (65). The β-value is the measure of DNA methylation for each individual CpG probe, where a minimum value of 0 indicates a fully unmethylated CpG and a maximum value of 1 indicates a fully methylated CpG in the population. CpG probes with a detection p-value > 0.05 in any one sample were excluded from the analysis. Differentially methylated regions (DMRs) were called using the Bioconductor package “DMRcate” (v3.0.10), and regions were considered differentially methylated if at least five contiguous CpGs demonstrated a mean difference of 0.20 methylation change in the drug treated cells compared to VEH treated RKO cells.

### Construction and sequencing of directional total RNA-seq libraries

Libraries were prepared by the Van Andel Institute Genomics Core (RRID:SCR_022913) from 500 ng of total RNA using the KAPA RNA HyperPrep Kit (Kapa Biosystems, Wilmington, MA USA). Ribosomal RNA material was reduced using the QIAseq FastSelect –rRNA HMR Kit (Qiagen, Germantown, MD, USA). RNA was sheared to 300-400 bp. Prior to PCR amplification, cDNA fragments were ligated to IDT for Illumina TruSeq UD Indexed adapters (Illumina Inc, San Diego CA, USA). Quality and quantity of the finished libraries were assessed using a combination of Agilent DNA High Sensitivity chip (Agilent Technologies, Inc.) and QuantiFluor^®^ dsDNA System (Promega Corp., Madison, WI, USA). Individually indexed libraries were pooled and 50 bp, paired end sequencing was performed on an Illumina NovaSeq6000 sequencer to an average depth of 50M raw paired-reads per transcriptome. Base calling was done by Illumina RTA3 and output of NCS was demultiplexed and converted to FastQ format with Illumina Bcl2fastq (v1.9.0).

### RNA-seq processing and analysis

Raw 50 bp paired-end reads were trimmed with TrimGalore! (http://www.bioinformatics.babraham.ac.uk/projects/trim_galore/) (RRID:SCR_011847) followed by quality control analysis with FastQC. Trimmed reads were aligned to GRCh38.p12 and indexed to GENCODE v29 via STAR (v2.5.3a) aligner with flags ‘-twopassMode Basic\ -quantMode GeneCounts’ for feature counting.

ReadsPerGene output count files were constructed into a raw read count matrix in R. Low count genes were filtered (1 count in at least one sample) prior to edgeR (v4.2.2) count normalization and differential expression analysis with voomWithQualityWeights and quasi-likelihood fit set to robust. Principal component analysis was calculated using ‘prcomp’ in the R stats package on the normalized expression matrix. Differential expression analysis was conducted with voomWithQualityWeights and a quasi-likelihood fit set to robust. Each dataset was treated separately for differential expression analysis, and all inhibitor treatments were compared to their respective vehicle samples. Genes were considered differentially expressed if ∣Log_2_FC∣ ≥ 1 and FDR ≤ 0.01.

### Gene Set Enrichment Analysis (GSEA)

GSEA (v4.3.3) (66) was conducted across the HALLMARK and c2.cp.kegg curated gene set databases from Molecular Signature Database (MSigDB). Phenotype comparisons were set to the sample of interest (e.g. VD) VS REST (single-agent treatments; e.g. VEH/DAC/VAL) for all analysis with weighted enrichment statistic and Signal2Noise settings for ranking genes. Maximum and minimum size of a gene set was set to 1500 and 15 genes, respectively. Genes marked as a “core enrichment” gene were used for epigenetic profiling and heatmap row z-score analysis of normalized cpm values.

### Integrative genomic analysis

As described in the respective sections above, bigwig files were generated for each sample for genome-wide H3K27ac and H3K27me1 efficiency (siQ-ChIP-seq), DNA methylation β-values (EPIC array), and publicly available datasets for RKO parental Whole Genome Bisulfite Sequencing (GSE262054, WGBS) (67) and our previously published H3K27me3 siQ-ChIP data (GSE256135) for parental and DNMT1i (GSK-3484862, 1 μM) treated RKO cells (16). Bed files with genomic coordinates for differential H3K27 peak analysis and ChromHMM chromatin state annotations were generated as described above. Integrated siQ-ChIP-seq, WGBS, and EPIC array analysis was conducted with deeptools (v3.2.0) (68) by constructing matrices with ‘computeMatrix’ across queried genomic coordinates with the respective bigwig data and visualizing the summarized integration with ‘plotProfile’ and ‘plotHeatmap’. Motif enrichment analysis was conducted using ‘findMotifsGenome.pl -len 8,10,12’ from HOMER (v4.11.1) (69).

### TCGA COADREAD patient data

Colorectal adenocarcinoma (COADREAD) TCGA patient data was accessed via the UCSC Xena Genome Browser (70). EZH1 and EZH2 normalized mRNA expression data was processed with patient survival outcomes using survival (https://github.com/therneau/survival, v3.8-3) and survminer (https://github.com/kassambara/survminer, v.0.5). Gene set enrichment analysis was conducted on patient samples using the Xena Browser plug-in for blizGSEA (https://github.com/MaayanLab/blitzgsea) (71).

### Data and Code Availability

All sequencing and EPIC array data will be made available on the Gene Expression Omnibus (GEO) upon acceptance of publication. A reviewer only token will be generated upon manuscript submission to a journal. Code for bioinformatic analysis will be deposited on our GitHub page and archived with Zenodo prior to submission to a journal.

### Recombinant nucleosome preparation

Recombinant mononucleosomes were reconstituted according to standard methods. Briefly, *Xenopus laevis* histones H2A, H2B, H3, and H4 were purchased from The Histone Source (Colorado State University). Lyophilized histones were resuspended in unfolding buffer (20 mM Tris-HCl pH 7.5, 7 M guanidine-HCl, and 10 mM DTT). Octamers were assembled by mixing histones H2A/H2B/H3/H4 and dialyzing into refolding buffer (10 mM Tris-HCl pH 7.5, 2 M NaCl, 1 mM EDTA, 5 mM BME) at 4°C, then purified by size exclusion chromatography using a Superdex 200 column (Cytiva). 175 bp DNA templates were generated by PCR amplification of the 147 NPS sequence and purified by ion exchange chromatography using a RESOURCE Q column (Cytiva). Octamers and DNA templates were mixed and assembled into nucleosomes via an 18 hour dialysis from RB high (10 mM Tris-HCl pH 7.5, 2 M KCl, 1 mM EDTA, and 1 mM DTT) to RB low (10 mM Tris-HCl pH 7.5, 250 mM KCl, 1 mM EDTA, and 1 mM DTT) at 4°C.

### *In vitro* PRC2 activity assays

EZH1 or EZH2-containing recombinant PRC2 complexes also comprising SUZ12, EED, RbAp46, and RbAp48 were purchased (Active Motif 31500 and 31387, respectively). PRC2 activity assays containing 20 nM PRC2 complex, 750 nM recombinant nucleosome, 20 μM SAM, and inhibitor or vehicle (DMSO) were assembled in reaction buffer (20 mM Tris-HCl pH 8, 50 mM NaCl, 1 mM EDTA, 3 mM MgCl_2_, and 0.1 mg/mL BSA) in a final volume of 10 μL. Reactions were incubated at room temperature overnight, then quenched by adding TFA to 0.5% final. PRC2 activity was measured using the MTase-Glo Methyltransferase Assay (Promega V7601) according to the manufacturer’s instructions. IC_50_ values were generated using GraphPad Prism (RRID:SCR_002798).

## Supplementary Material

Supplement 1

## Highlights

- Dual EZH1/2 inhibitors reduce all three H3K27 methylation states and robustly synergize with DNMT inhibitors.
- EZH1 mediates H3K27me1 deposition at deeply Polycomb-marked regions in the absence of EZH2.
- EZH inhibitors induce a bivalent repressive state marked by DNA methylation and H3K27ac that limits p300/CBP-dependent transcription.
- DNMT inhibitor-induced “epigenetic switching” is more vulnerable to dual EZH1/2 blockade than selective EZH2 inhibition.
- Combined EZH1/2 and DNMT inhibition redistributes H3K27ac away from pro-growth oncogenic signaling genes and towards bivalent genes.
- Suppression of oncogenic signaling by dual DNMTi and EZH1/2 inhibition is a key efficacy signature of this drug combination.

## References

[1] Hanahan D. Hallmarks of Cancer: New Dimensions. Cancer Discov. 2022;12:31–46. 35022204 10.1158/2159-8290.CD-21-1059

[2] Esteller M, Dawson MA, Kadoch C, Rassool FV, Jones PA, Baylin SB. The Epigenetic Hallmarks of Cancer. Cancer Discov. 2024;14:1783–809. 39363741 10.1158/2159-8290.CD-24-0296

[3] Gore SD, Jones C, Kirkpatrick P. Decitabine. Nat Rev Drug Discov. Nature Publishing Group; 2006;5:891–2. 17117522 10.1038/nrd2180

[4] Issa J-P, Kantarjian H. Azacitidine. Nat Rev Drug Discov. 2005;Suppl:S6–7. 15962522 10.1038/nrd1726

[5] Feng S, De Carvalho DD. Clinical advances in targeting epigenetics for cancer therapy. FEBS J. 2022;289:1214–39. 33545740 10.1111/febs.15750

[6] Lee H-Z, Kwitkowski VE, Del Valle PL, Ricci MS, Saber H, Habtemariam BA, FDA Approval: Belinostat for the Treatment of Patients with Relapsed or Refractory Peripheral T-cell Lymphoma. Clin Cancer Res Off J Am Assoc Cancer Res. 2015;21:2666–70. 10.1158/1078-0432.CCR-14-3119 25802282

[7] Pu J, Liu T, Wang X, Sharma A, Schmidt-Wolf IGH, Jiang L, Exploring the role of histone deacetylase and histone deacetylase inhibitors in the context of multiple myeloma: mechanisms, therapeutic implications, and future perspectives. Exp Hematol Oncol. 2024;13:45. 38654286 10.1186/s40164-024-00507-5 PMC11040994

[8] Rothbart SB, Baylin SB. Epigenetic Therapy for Epithelioid Sarcoma. Cell. 2020;181:211. 32302562 10.1016/j.cell.2020.03.042

[9] Morin RD, Arthur SE, Assouline S. Treating lymphoma is now a bit EZ-er. Blood Adv. 2021;5:2256–63. 33904892 10.1182/bloodadvances.2020002773 PMC8095133

[10] Diesch J, Zwick A, Garz A-K, Palau A, Buschbeck M, Götze KS. A clinical-molecular update on azanucleoside-based therapy for the treatment of hematologic cancers. Clin Epigenetics. 2016;8:71. 27330573 10.1186/s13148-016-0237-y PMC4915187

[11] Dai W, Qiao X, Fang Y, Guo R, Bai P, Liu S, Epigenetics-targeted drugs: current paradigms and future challenges. Signal Transduct Target Ther. Nature Publishing Group; 2024;9:332. 39592582 10.1038/s41392-024-02039-0 PMC11627502

[12] Kim N, Norsworthy KJ, Subramaniam S, Chen H, Manning ML, Kitabi E, FDA Approval Summary: Decitabine and Cedazuridine Tablets for Myelodysplastic Syndromes. Clin Cancer Res. 2022;28:3411–6. 35435961 10.1158/1078-0432.CCR-21-4498 PMC9378483

[13] Pappalardi MB, Keenan K, Cockerill M, Kellner WA, Stowell A, Sherk C, Discovery of a first-in-class reversible DNMT1-selective inhibitor with improved tolerability and efficacy in acute myeloid leukemia. Nat Cancer. Nature Publishing Group; 2021;2:1002–17. 34790902 PMC8594913

[14] Gilmartin AG, Groy A, Gore ER, Atkins C, Long ER, Montoute MN, In vitro and in vivo induction of fetal hemoglobin with a reversible and selective DNMT1 inhibitor. Haematologica. 2020; 10.3324/haematol.2020.248658 PMC8252945 32586904

[15] Chomiak AA, Tiedemann RL, Liu Y, Kong X, Cui Y, Wiseman AK, Select EZH2 inhibitors enhance viral mimicry effects of DNMT inhibition through a mechanism involving NFAT:AP-1 signaling. Sci Adv. 2024;10:eadk4423. 38536911 10.1126/sciadv.adk4423 PMC10971413

[16] Liu Y, Hrit JA, Chomiak AA, Stransky S, Hoffman JR, Tiedemann RL, DNA hypomethylation promotes UHRF1-and SUV39H1/H2-dependent crosstalk between H3K18ub and H3K9me3 to reinforce heterochromatin states. Mol Cell. 2024;S1097-2765(24)00914-6. 10.1016/j.molcel.2024.11.009 PMC11741932 39631394

[17] Deblois G, Tonekaboni SAM, Grillo G, Martinez C, Kao YI, Tai F, Epigenetic Switch-Induced Viral Mimicry Evasion in Chemotherapy-Resistant Breast Cancer. Cancer Discov. 2020;10:1312–29. 32546577 10.1158/2159-8290.CD-19-1493

[18] Dimopoulos K, Helbo AS, Munch-Petersen HF, Sjö L, Christensen J, Kristensen LS, Dual inhibition of DNMTs and EZH2 can overcome both intrinsic and acquired resistance of myeloma cells to IMiDs in a cereblon-independent manner. Mol Oncol. 2018;12:180–95. 29130642 10.1002/1878-0261.12157 PMC5792743

[19] Egger G, Jeong S, Escobar SG, Cortez CC, Li TWH, Saito Y, Identification of DNMT1 (DNA methyltransferase 1) hypomorphs in somatic knockouts suggests an essential role for DNMT1 in cell survival. Proc Natl Acad Sci U S A. 2006;103:14080–5. 16963560 10.1073/pnas.0604602103 PMC1599915

[20] Izutsu K, Makita S, Nosaka K, Yoshimitsu M, Utsunomiya A, Kusumoto S, An open-label, single-arm phase 2 trial of valemetostat for relapsed or refractory adult T-cell leukemia/lymphoma. Blood. 2023;141:1159–68. 36150143 10.1182/blood.2022016862 PMC10651775

[21] Morschhauser F, Tilly H, Chaidos A, McKay P, Phillips T, Assouline S, Tazemetostat for patients with relapsed or refractory follicular lymphoma: an open-label, single-arm, multicentre, phase 2 trial. Lancet Oncol. Elsevier; 2020;21:1433–42. 33035457 10.1016/S1470-2045(20)30441-1 PMC8427481

[22] Honma D, Kanno O, Watanabe J, Kinoshita J, Hirasawa M, Nosaka E, Novel orally bioavailable EZH1/2 dual inhibitors with greater antitumor efficacy than an EZH2 selective inhibitor. Cancer Sci. 2017;108:2069–78. 28741798 10.1111/cas.13326 PMC5623739

[23] Keller PJ, Adams EJ, Wu R, Côté A, Arora S, Cantone N, Comprehensive Target Engagement by the EZH2 Inhibitor Tulmimetostat Allows for Targeting of ARID1A Mutant Cancers. Cancer Res. 2024;84:2501–17. 38833522 10.1158/0008-5472.CAN-24-0398 PMC11292196

[24] Porazzi P, Nason S, Yang Z, Carturan A, Ghilardi G, Guruprasad P, EZH1/EZH2 inhibition enhances adoptive T cell immunotherapy against multiple cancer models. Cancer Cell. Elsevier; 2025;43:537–551.e7. 39983725 10.1016/j.ccell.2025.01.013 PMC13312562

[25] Shinohara H, Sawado R, Nakagawa M, Hattori A, Yamagata K, Tauchi K, Dual targeting of EZH1 and EZH2 for the treatment of malignant rhabdoid tumors. Mol Ther - Oncolytics. Elsevier; 2022;27:14–25. 36212776 10.1016/j.omto.2022.09.006 PMC9529991

[26] Wang J, Yu X, Gong W, Liu X, Park K-S, Ma A, EZH2 noncanonically binds cMyc and p300 through a cryptic transactivation domain to mediate gene activation and promote oncogenesis. Nat Cell Biol. 2022;24:384–99. 35210568 10.1038/s41556-022-00850-x PMC9710513

[27] Shen X, Liu Y, Hsu Y-J, Fujiwara Y, Kim J, Mao X, EZH1 Mediates Methylation on Histone H3 Lysine 27 and Complements EZH2 in Maintaining Stem Cell Identity and Executing Pluripotency. Mol Cell. 2008;32:491–502. 19026780 10.1016/j.molcel.2008.10.016 PMC2630502

[28] Tagami H, Ray-Gallet D, Almouzni G, Nakatani Y. Histone H3.1 and H3.3 Complexes Mediate Nucleosome Assembly Pathways Dependent or Independent of DNA Synthesis. Cell. Elsevier; 2004;116:51–61. 14718166 10.1016/s0092-8674(03)01064-x

[29] Wu RS, Tsai S, Bonner WM. Patterns of histone variant synthesis can distinguish G0 from G1 cells. Cell. 1982;31:367–74. 7159927 10.1016/0092-8674(82)90130-1

[30] Goldberg AD, Banaszynski LA, Noh K-M, Lewis PW, Elsaesser SJ, Stadler S, Distinct factors control histone variant H3.3 localization at specific genomic regions. Cell. 2010;140:678–91. 20211137 10.1016/j.cell.2010.01.003 PMC2885838

[31] Dickson BM, Tiedemann RL, Chomiak AA, Cornett EM, Vaughan RM, Rothbart SB. A physical basis for quantitative ChIP-sequencing. J Biol Chem. 2020;295:15826–37. 32994221 10.1074/jbc.RA120.015353 PMC7681007

[32] Dickson BM, Kupai A, Vaughan RM, Rothbart SB. Streamlined quantitative analysis of histone modification abundance at nucleosome-scale resolution with siQ-ChIP version 2.0. Sci Rep. 2023;13:7508. 37160995 10.1038/s41598-023-34430-2 PMC10169836

[33] Ferrari KJ, Scelfo A, Jammula S, Cuomo A, Barozzi I, Stützer A, Polycomb-Dependent H3K27me1 and H3K27me2 Regulate Active Transcription and Enhancer Fidelity. Mol Cell. 2014;53:49–62. 24289921 10.1016/j.molcel.2013.10.030

[34] Cui K, Zang C, Roh T-Y, Schones DE, Childs RW, Peng W, Chromatin Signatures in Multipotent Human Hematopoietic Stem Cells Indicate the Fate of Bivalent Genes during Differentiation. Cell Stem Cell. 2009;4:80–93. 19128795 10.1016/j.stem.2008.11.011 PMC2785912

[35] Qu X, Liang Y, McCornack C, Xing X, Schmidt H, Tomlinson C, Charting the regulatory landscape of TP53 on transposable elements in cancer. Genome Res. 2025;35:1456–71. 40360186 10.1101/gr.279398.124 PMC12129084

[36] Ai S, Yu X, Li Y, Peng Y, Li C, Yue Y, Divergent Requirements for EZH1 in Heart Development Versus Regeneration. Circ Res. American Heart Association; 2017;121:106–12. 28512107 10.1161/CIRCRESAHA.117.311212 PMC5527745

[37] Xu J, Shao Z, Li D, Xie H, Kim W, Huang J, Developmental control of polycomb subunit composition by GATA factors mediates a switch to non-canonical functions. Mol Cell. 2015;57:304–16. 25578878 10.1016/j.molcel.2014.12.009 PMC4305004

[38] Vo LT, Kinney MA, Liu X, Zhang Y, Barragan J, Sousa PM, Regulation of embryonic haematopoietic multipotency by EZH1. Nature. 2018;553:506–10. 29342143 10.1038/nature25435 PMC5785461

[39] Mochizuki-Kashio M, Aoyama K, Sashida G, Oshima M, Tomioka T, Muto T, Ezh2 loss in hematopoietic stem cells predisposes mice to develop heterogeneous malignancies in an Ezh1-dependent manner. Blood. 2015;126:1172–83. 26219303 10.1182/blood-2015-03-634428

[40] Koeppel M, van Heeringen SJ, Kramer D, Smeenk L, Janssen-Megens E, Hartmann M, Crosstalk between c-Jun and TAp73alpha/beta contributes to the apoptosis-survival balance. Nucleic Acids Res. 2011;39:6069–85. 21459846 10.1093/nar/gkr028 PMC3152320

[41] McLean CY, Bristor D, Hiller M, Clarke SL, Schaar BT, Lowe CB, GREAT improves functional interpretation of cis-regulatory regions. Nat Biotechnol. 2010;28:495–501. 20436461 10.1038/nbt.1630 PMC4840234

[42] Mousavi K, Zare H, Wang AH, Sartorelli V. Polycomb protein Ezh1 promotes RNA polymerase II elongation. Mol Cell. 2012;45:255–62. 22196887 10.1016/j.molcel.2011.11.019 PMC12310276

[43] Liu P, Nadeef S, Serag MF, Paytuví-Gallart A, Abadi M, Della Valle F, PRC2-EZH1 contributes to circadian gene expression by orchestrating chromatin states and RNA polymerase II complex stability. EMBO J. 2024;43:6052–75. 39433902 10.1038/s44318-024-00267-2 PMC11612306

[44] Neri F, Rapelli S, Krepelova A, Incarnato D, Parlato C, Basile G, Intragenic DNA methylation prevents spurious transcription initiation. Nature. 2017;543:72–7. 28225755 10.1038/nature21373

[45] Yang X, Han H, De Carvalho DD, Lay FD, Jones PA, Liang G. Gene Body Methylation Can Alter Gene Expression and Is a Therapeutic Target in Cancer. Cancer Cell. 2014;26:577–90. 25263941 10.1016/j.ccr.2014.07.028 PMC4224113

[46] Feng S, De Carvalho DD. Clinical advances in targeting epigenetics for cancer therapy. FEBS J. 2022;289:1214–39. 33545740 10.1111/febs.15750

[47] Reddy D, Bhattacharya S, Workman JL. (mis)-Targeting of SWI/SNF complex(es) in cancer. Cancer Metastasis Rev. 2023;42:455–70. 37093326 10.1007/s10555-023-10102-5 PMC10349013

[48] McCole R, Nolan J, Reck DM, Monger C, Rustichelli S, Conway E, A conserved switch to less catalytically active Polycomb repressive complexes in non-dividing cells. Cell Rep. 2025;44:115192. 39799569 10.1016/j.celrep.2024.115192 PMC11931288

[49] Ran FA, Hsu PD, Wright J, Agarwala V, Scott DA, Zhang F. Genome engineering using the CRISPR-Cas9 system. Nat Protoc. 2013;8:2281–308. 24157548 10.1038/nprot.2013.143 PMC3969860

[50] Wiederschain D, Wee S, Chen L, Loo A, Yang G, Huang A, Single-vector inducible lentiviral RNAi system for oncology target validation. Cell Cycle Georget Tex. 2009;8:498–504. 10.4161/cc.8.3.7701 19177017

[51] Wee S, Wiederschain D, Maira S-M, Loo A, Miller C, deBeaumont R, PTEN-deficient cancers depend on PIK3CB. Proc Natl Acad Sci. Proceedings of the National Academy of Sciences; 2008;105:13057–62. 10.1073/pnas.0802655105 PMC2529105 18755892

[52] Arafeh R, Shibue T, Dempster JM, Hahn WC, Vazquez F. The present and future of the Cancer Dependency Map. Nat Rev Cancer. Nature Publishing Group; 2025;25:59–73. 39468210 10.1038/s41568-024-00763-x

[53] Livak KJ, Schmittgen TD. Analysis of Relative Gene Expression Data Using Real-Time Quantitative PCR and the 2-ΔΔCT Method. Methods. 2001;25:402–8. 11846609 10.1006/meth.2001.1262

[54] Rothbart SB, Dickson BM, Raab JR, Grzybowski AT, Krajewski K, Guo AH, An Interactive Database for the Assessment of Histone Antibody Specificity. Mol Cell. 2015;59:502–11. 26212453 10.1016/j.molcel.2015.06.022 PMC4530063

[55] Sidoli S, Bhanu NV, Karch KR, Wang X, Garcia BA. Complete Workflow for Analysis of Histone Post-translational Modifications Using Bottom-up Mass Spectrometry: From Histone Extraction to Data Analysis. J Vis Exp JoVE. 2016;54112. 27286567 10.3791/54112 PMC4927705

[56] Joseph-Chowdhury J-SN, Stransky S, Graff S, Cutler R, Young D, Kim JS, Global level quantification of histone post-translational modifications in a 3D cell culture model of hepatic tissue. J Vis Exp JoVE. 2022; 10.3791/63606. PMC10042382 35604167

[57] Yuan Z-F, Sidoli S, Marchione DM, Simithy J, Janssen KA, Szurgot MR, EpiProfile 2.0: A Computational Platform for Processing Epi-Proteomics Mass Spectrometry Data. J Proteome Res. American Chemical Society; 2018;17:2533–41. 29790754 10.1021/acs.jproteome.8b00133 PMC6387837

[58] Cornett EM, Dickson BM, Rothbart SB. Analysis of Histone Antibody Specificity with Peptide Microarrays. J Vis Exp JoVE. 2017;55912. 28809825 10.3791/55912 PMC5613814

[59] Dickson BM, Cornett EM, Ramjan Z, Rothbart SB. ArrayNinja: An Open Source Platform for Unified Planning and Analysis of Microarray Experiments. Methods Enzymol. 2016;574:53–77. 27423857 10.1016/bs.mie.2016.02.002 PMC5353857

[60] Faust GG, Hall IM. SAMBLASTER: fast duplicate marking and structural variant read extraction. Bioinforma Oxf Engl. 2014;30:2503–5. 10.1093/bioinformatics/btu314 PMC4147885 24812344

[61] Scrucca L, Fraley C, Murphy TB, Raftery AE. Model-Based Clustering, Classification, and Density Estimation Using mclust in R. New York: Chapman and Hall/CRC; 2023.

[62] Ernst J, Kellis M. ChromHMM: automating chromatin-state discovery and characterization. Nat Methods. Nature Publishing Group; 2012;9:215–6. 22373907 10.1038/nmeth.1906 PMC3577932

[63] Bibikova M, Le J, Barnes B, Saedinia-Melnyk S, Zhou L, Shen R, Genome-wide DNA methylation profiling using Infinium^®^ assay. Epigenomics. 2009;1:177–200. 22122642 10.2217/epi.09.14

[64] Bibikova M, Barnes B, Tsan C, Ho V, Klotzle B, Le JM, High density DNA methylation array with single CpG site resolution. Genomics. 2011;98:288–95. 21839163 10.1016/j.ygeno.2011.07.007

[65] Zhou W, Triche TJ, Laird PW, Shen H. SeSAMe: reducing artifactual detection of DNA methylation by Infinium BeadChips in genomic deletions. Nucleic Acids Res. 2018;46:e123. 30085201 10.1093/nar/gky691 PMC6237738

[66] Subramanian A, Tamayo P, Mootha VK, Mukherjee S, Ebert BL, Gillette MA, Gene set enrichment analysis: a knowledge-based approach for interpreting genome-wide expression profiles. Proc Natl Acad Sci U S A. 2005;102:15545–50. 16199517 10.1073/pnas.0506580102 PMC1239896

[67] Qu X, Liang Y, McCornack C, Xing X, Schmidt H, Tomlinson C, Charting the regulatory landscape of TP53 on transposable elements in cancer. Genome Res. 2025;35:1456–71. 40360186 10.1101/gr.279398.124 PMC12129084

[68] Ramírez F, Dündar F, Diehl S, Grüning BA, Manke T. deepTools: a flexible platform for exploring deepsequencing data. Nucleic Acids Res. 2014;42:W187–191. 24799436 10.1093/nar/gku365 PMC4086134

[69] Heinz S, Benner C, Spann N, Bertolino E, Lin YC, Laslo P, Simple combinations of lineage-determining transcription factors prime cis-regulatory elements required for macrophage and B cell identities. Mol Cell. 2010;38:576–89. 20513432 10.1016/j.molcel.2010.05.004 PMC2898526

[70] Muzny DM, Bainbridge MN, Chang K, Dinh HH, Drummond JA, Fowler G, Comprehensive molecular characterization of human colon and rectal cancer. Nature. Nature Publishing Group; 2012;487:330–7. 22810696 10.1038/nature11252 PMC3401966

[71] Lachmann A, Xie Z, Ma’ayan A. blitzGSEA: efficient computation of gene set enrichment analysis through gamma distribution approximation. Bioinforma Oxf Engl. 2022;38:2356–7. 10.1093/bioinformatics/btac076 PMC9004650 35143610

