# Supplementary material for: Dual EZH1/2 inhibition enhances DNMT inhibitor efficacy in colon cancer through targeting H3K27me1
Source: bioRxiv. 2025 Sep 18:2025.09.16.676613. Preprint. [Version 1] doi: 10.1101/2025.09.16.676613 (PMC12458120; doi:10.1101/2025.09.16.676613)
Supplement: Supplement 1 [file media-1.pdf]

Supplemental Information

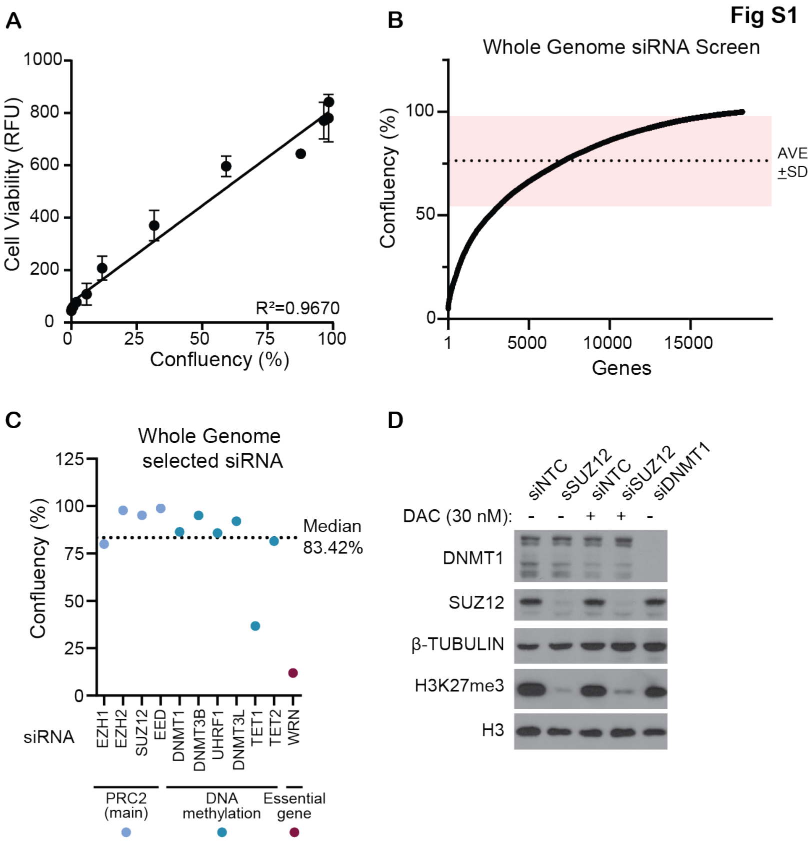

Figure S1. Genome-wide siRNA screen optimization and validation

A) CellTiter-Fluor cell viability (Relative fluorescence units, RFU) compared to cell confluency (%) determined from brightfield images 72 hours following titration of wild-type HCT116 cell number.

B) Cell confluency (%) determined from brightfield images of each well transfected with siRNA pools and used to normalize NLuc signal in genome-wide siRNA screen from Figure 1B. Genes are ordered from low to high confluency. Dotted line represents average screen confluency, and pink band represents ±SD.

C) Confluency (%) of selected epigenetic genes of interest from Figure 1D compared to knockdown of *WRN*, an essential HCT116 gene according to DEPMAP CRISPR and RNAi screens.

D) Western blot analysis of knockdown efficiency and H3K27me3 levels from wild-type HCT116 cells following 72-hour siRNA transfection with or without DAC corresponding to samples in Figure 1E.

Fig S2

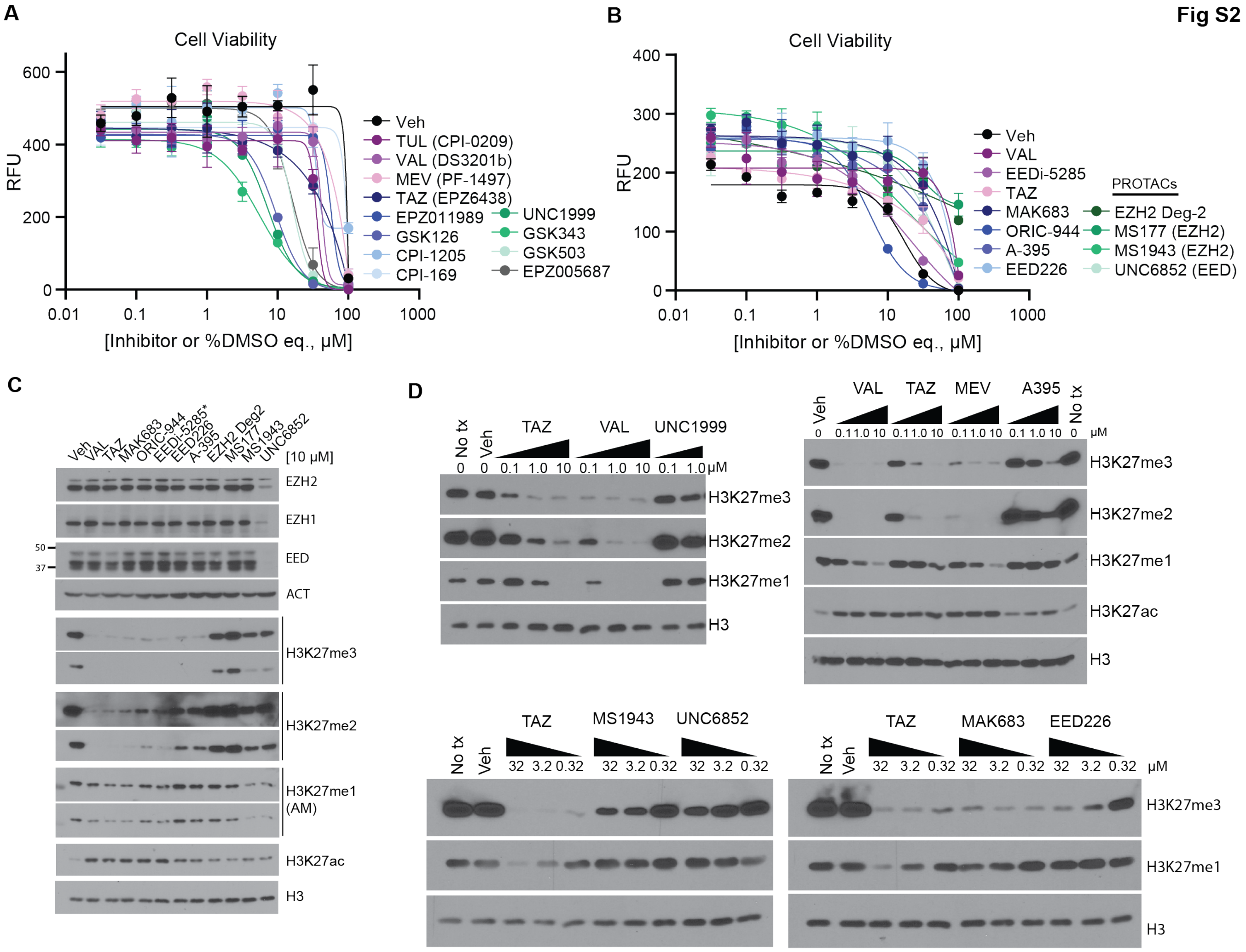

Figure S2. Dual EZH1/2 inhibitors block all three H3K27 methylation states

A-B) Dose response cell viability curves (CellTiter-Fluor relative fluorescence units, RFU) for the *SFRP1*-NLuc Reporter HCT116 cells exposed to the indicated SAM-competitive EZH inhibitors from Figure 1A **(A)** or all other PRC2i compared to the SAM-competitive EZH inhibitors TAZ and VAL from Figure 1B **(B)** for 72 hours. Data are representative of three biological replicates, and the mean ±SD of technical triplicates from a single experiment is shown.

C) Western blot analysis with short and long exposures of all H3K27 modification states from wild-type HCT116 cells following 72-hour exposure to molecules in Figure 1B, including vehicle (DMSO, % equivalent), EED inhibitors, or subunit PROTACs compared to SAM-competitive EZH inhibitors TAZ and VAL. All molecules were used at 10 μM and ordered approximately by efficacy in the *SFRP1*-NLuc assay. *Indicates where order differs from Figure 1C where molecules were used and ordered by efficacy at 1 μM.

D) Western blot analysis of all H3K27 methylation states from wild-type HCT116 cells following 72-hour exposure to vehicle (DMSO, % equivalent) or titrations of PRC2 inhibitors. VAL: valemetostat, MEV: mevrometostat, and TAZ: tazemetostat.

Fig S3

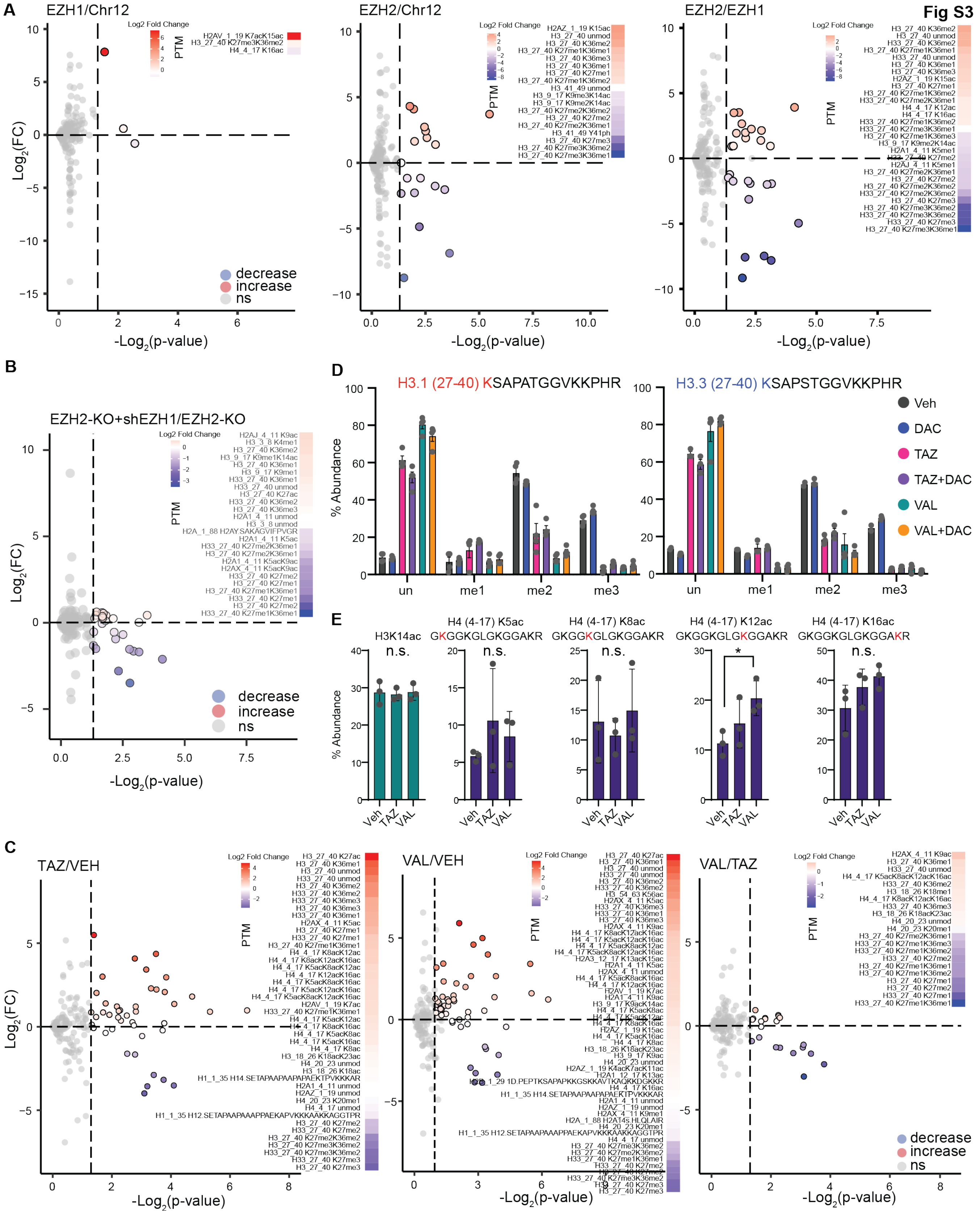

A
EZH1/Chr12
EZH2/Chr12
EZH2/EZH1
Log2 Fold Change
PTM
Log2(FC)
-Log2(p-value)
decrease
increase
ns
B
EZH2-KO+shEZH1/EZH2-KO
C
TAZ/VEH
VAL/VEH
VAL/TAZ
D
H3.1 (27-40) KSAPATGGVKKPHR
H3.3 (27-40) KSAPSTGGVKKPHR
% Abundance
un
me1
me2
me3
Veh
DAC
TAZ
TAZ+DAC
VAL
VAL+DAC
E
H3K14ac
H4 (4-17) K5ac
GKGGKGLGKGGAKR
H4 (4-17) K8ac
GKGGKGLGKGGAKR
H4 (4-17) K12ac
GKGGKGLGKGGAKR
H4 (4-17) K16ac
GKGGKGLGKGGAKR
n.s.
*
Veh
TAZ
VAL

Figure S3. Mass spectrometry analysis of histone methylation states following EZH1/2 loss or inhibition

A) Volcano plots showing differential histone modifications between EZH1 CRISPR-KO or EZH2 CRISPR-KO and Chr12 CRISPR controls. Red indicates significant upregulation and blue indicates significant downregulation. Only significantly up- and down- regulated PTMs are indicated in the heat map labels. Each data point is derived from the fold change (FC) using the average abundances of three biological replicate clones for each CRISPR target from Figure 3B-C.

B) Volcano plot showing differential histone modifications between EZH2 CRISPR-KO and EZH2 CRISPR-KO + shEZH1. Each data point is derived from the fold change (FC) using the average abundances of three biological replicates from Figure 3E-F.

C) Volcano plot showing differential histone modifications between RKO cells treated with TAZ (1 μM), VAL (1 μM), or vehicle (DMSO, % equivalent) for 72 hours. Each data point is derived from the fold change (FC) using the average abundances of three biological replicates from Figure 3H.

D-E) Histone PTM mass spectrometry relative abundances of **D)** H3K27 methylation states of H3.1 and H3.3 or **E)** histone acetylation marks H3K18ac, H4K5ac, H4K8ac, H4K12ac, and H4K16ac in RKO cells treated with the EZH2i tazemetostat (TAZ, 1 μM) or EZH1/2i valemetostat (VAL, 1 μM) with or without DAC (300 nM) for 72 hours. Data are mean ± SD for biological triplicates.

Statistical significance was calculated using multiple unpaired T-tests. *$p<0.05$, **$p<0.01$, ***$p<0.001$

Fig S4

A

Mark	Company	ID	Lot	Concentration	Array Specificity
H3K27ac	ActiveMotif	39133	31521015	1 mg/mL	GOOD
H3K27me3	Cell Signaling, Rab mAb	9733S	19	0.102 mg/mL	GOOD
H3K27me1	ActiveMotif, Ms mAb	61015	3822017	1 mg/mL	GOOD
H3K27me1	AbClonal, Rab pAb	A2361	5500013107	0.52 mg/mL	GOOD
H3K27me1	Invitrogen, Rab pAb	712817	2349327	0.5 mg/mL	GOOD
H3K27me1	AbClonal, Rab pAb	A2361	5500022675	0.48 mg/mL	BAD
H3K27me1	Invitrogen, Rab pAb	712817	2532252	0.5 mg/mL	BAD

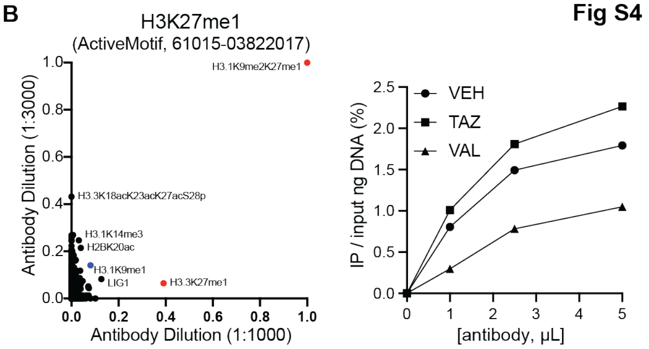

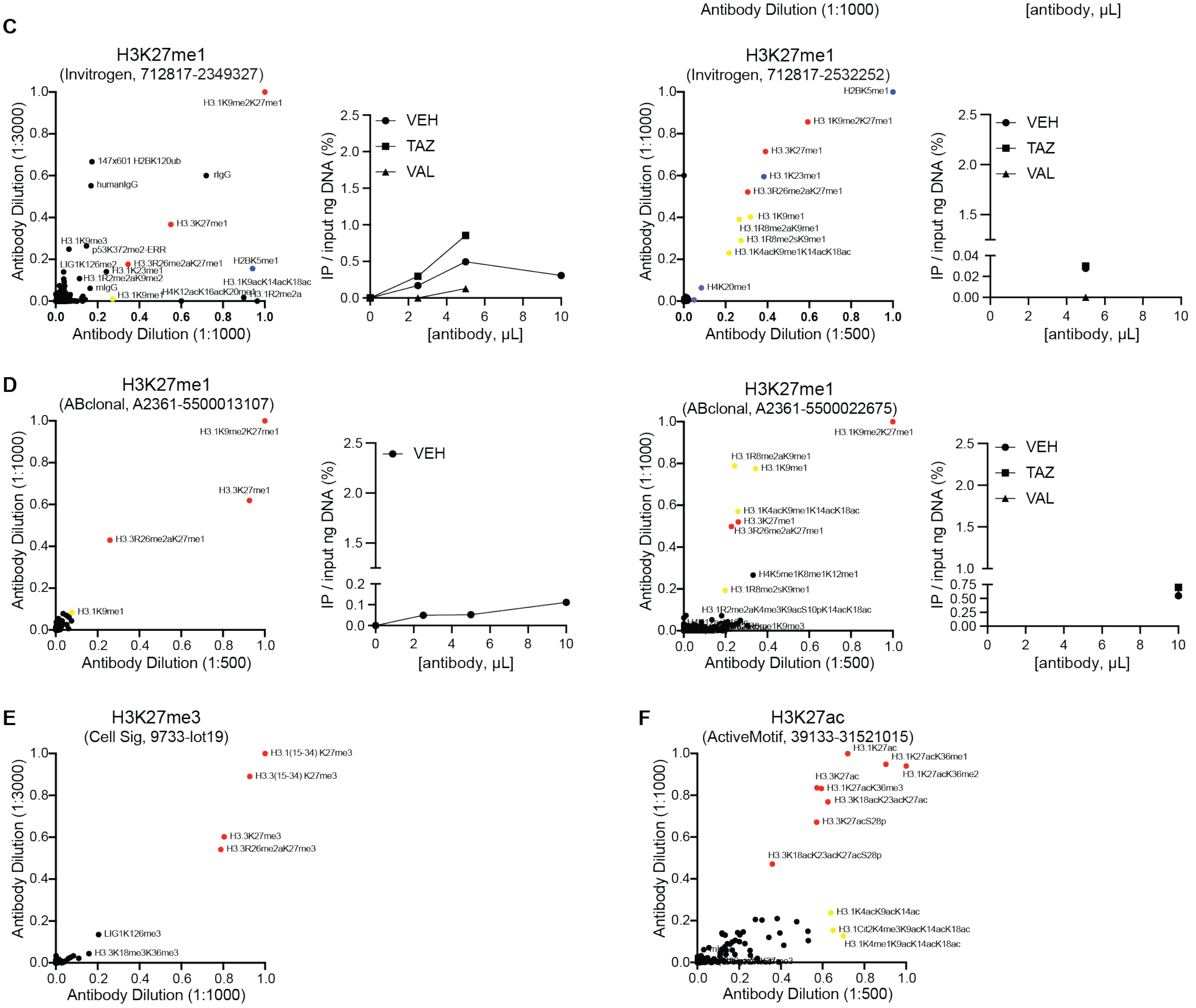

Figure S4. Antibody validation for ChIP

A) Antibodies tested for specificity and efficiency for use in ChIP in this study, ordered from best to worst.

B-F) Scatterplot from histone PTM peptide array (left) and antibody titration represented as % DNA immunoprecipitated from input in RKO cells (right) from the (B) H3K27me1 ActiveMotif

antibody, (C) H3K27me1 Invitrogen antibody, (D) H3K27me1 ABClonal antibody, (E) H3K27me3 Cell Signaling antibody, and (F) H3K27ac ActiveMotif antibody.

Fig S5

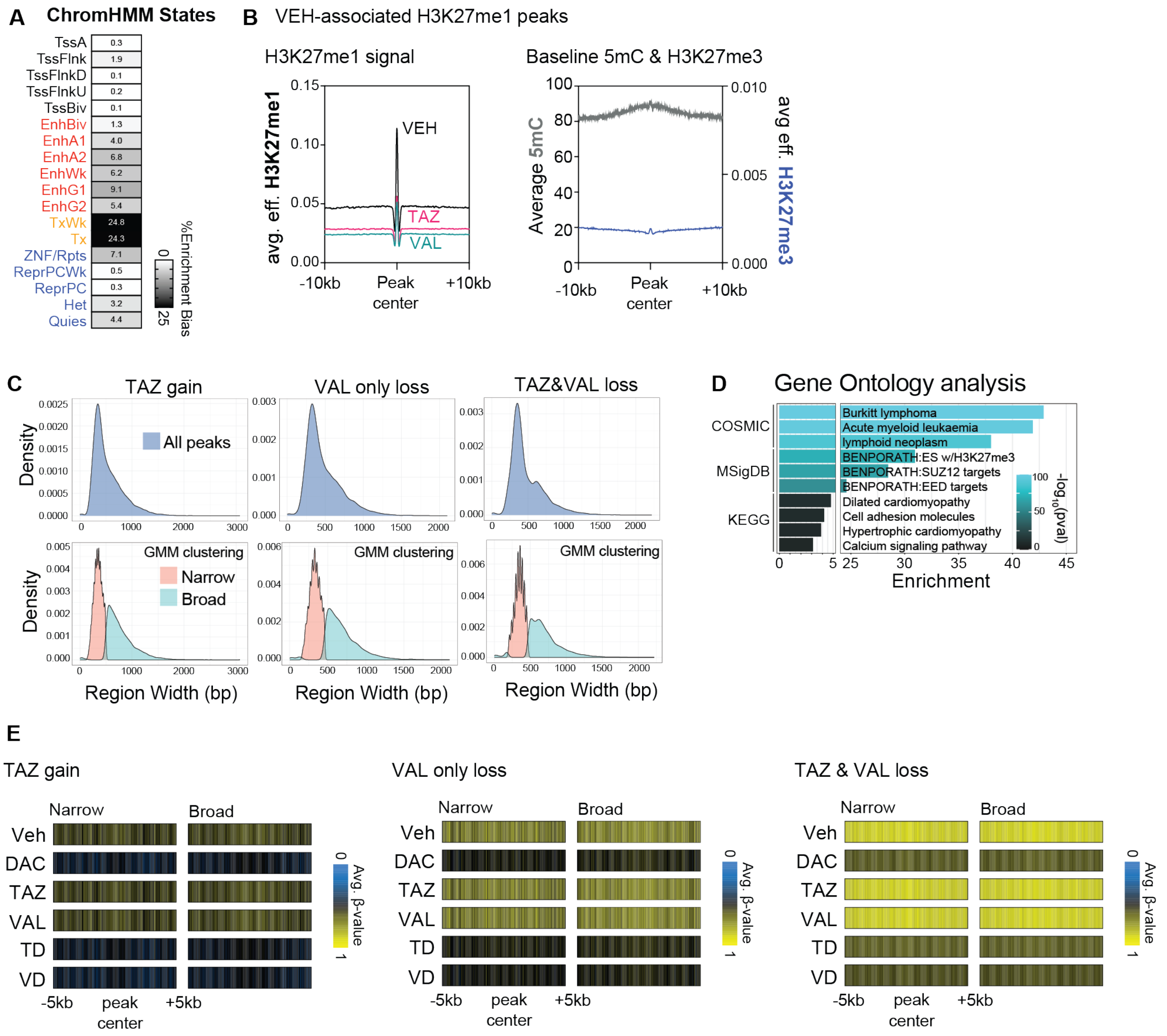

Figure S5. Baseline H3K27me1 distribution summary and DNA methylation among drug treatments.

A) Relative enrichment bias for VEH-associated H3K27me1 distributions across chromHMM states.

B) Average epigenetic profiles for VEH-treated H3K27me1 distributions (left) and baseline 5mC and H3K27me3 average levels (right) in RKO cells.

C) H3K27me1 distributions among TAZ-associated gains, VAL only associated loss, and shared TAZ and VAL used in Figure 4A. Top: Density distribution of all peaks. Bottom: Gaussian Mixture Modeling of H3K27me1 distributions into narrow and broad peaks.

D) Gene ontology analysis for TAZ-associated H3K27me1 gains.

E) Average DNA methylation profiles for each drug treatment centered on H3K27me1 altered peaks for TAZ-associated H3K27me1 gains (left), VAL only associated H3K27me1 loss (middle), and shared TAZ and VAL H3K27me1 loss (right).

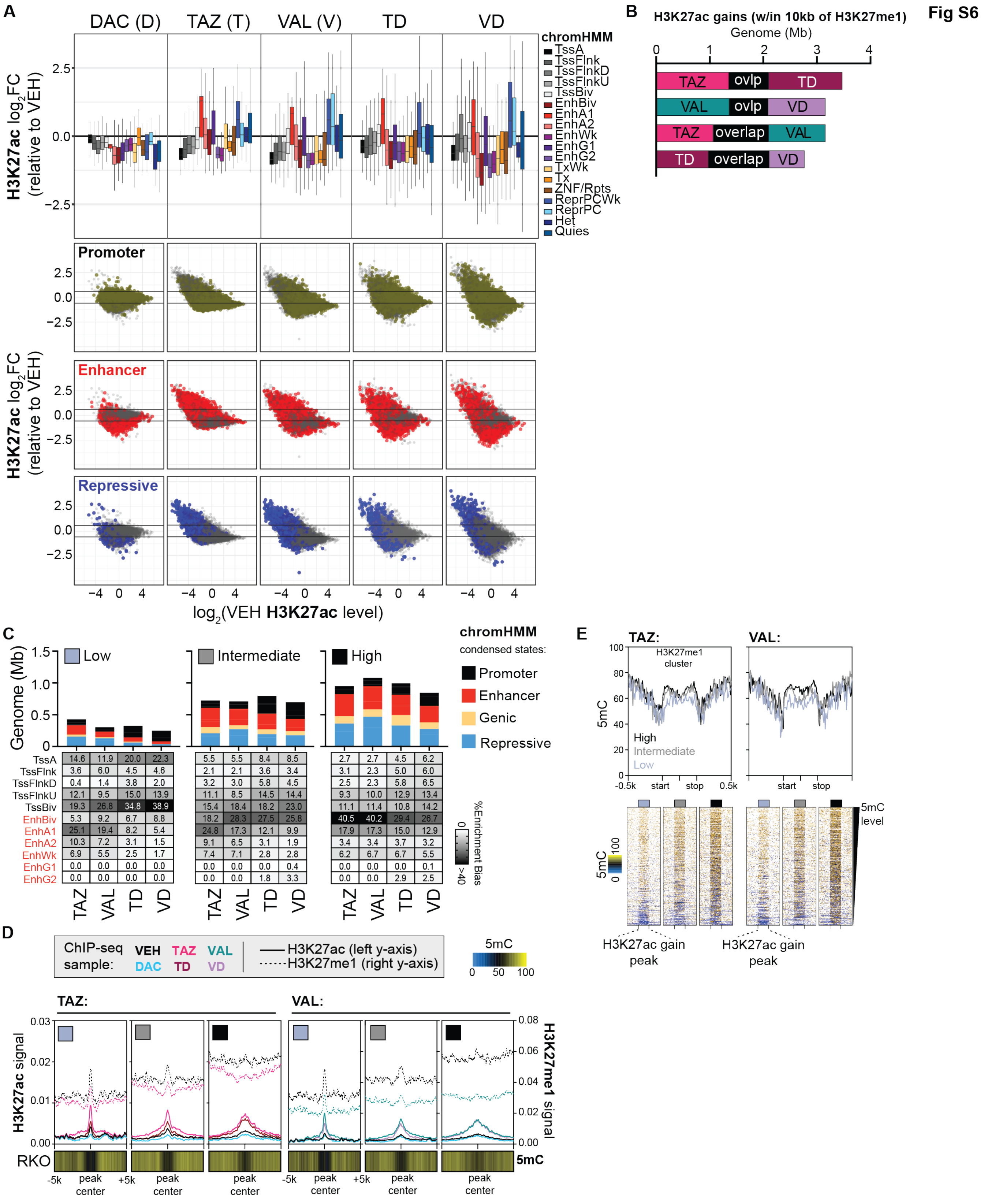

Figure S6. Characterization of H3K27ac genome dynamics across EZHi and EZHi/DNMTi

drug treatments.
A) Dynamics of H3K27ac change across drug treatments and classified by chromHMM state in RKO cells. Top: Boxplot of change in all H3K27ac peaks separated by individual chromHMM states. Outliers removed for figure clarity. Bottom: MA plots for each drug treatment, colored by chromHMM condensed state (Promoter, Enhancer, Repressive). Note, each MA plot for an individual drug treatment is the same but colored differently to highlight condensed chromHMM state. The x-axis represents the log2 transformation of H3K27ac signal in VEH, left to right can be interpreted as low to high H3K27ac levels in VEH-treated samples.
B) Stacked bar graph of H3K27ac gains genomic coverage (Mbs) among drug treatments. Overlap (ovlp) between genomic regions is indicated by black.
C) chromHMM characterization of H3K27ac gains clustered H3K27me1 signal. Top: Genomic coverage (in Mbs) of H3K27ac gains clustered by H3K27me1 (kmeans 3). Bottom: Relative enrichment bias for altered H3K27ac gained distributions across chromHMM states (note: Genic and Repressive chromHMM states omitted due to lack of enrichment and clarity of figure).
D) Epigenetic profiling of H3K27ac gains in EZHi treatments, clustered by H3K27me1. Top: Average H3K27ac (left y-axis) and H3K27me1 (right y-axis) siQ-ChIP efficiency centered on EZHi/DNMTi associated H3K27ac gains. Bottom: Baseline DNA methylation in RKO cells centered on H3K27ac gains.
E) DNA methylation analysis within scaled H3K27ac gained peaks (from Figure 5B) in single-agent EZHi treatments, clustered by H3K27me1. Top: Average DNA methylation profiles (WGBS) in parental RKO cells (GSE262054). Bottom: Heatmap of DNA methylation (WGBS) across individual H3K27ac gained peaks, sorted from the peak with the highest DNA methylation level to the lowest.
TD: TAZ + DAC, VD: VAL + DAC.

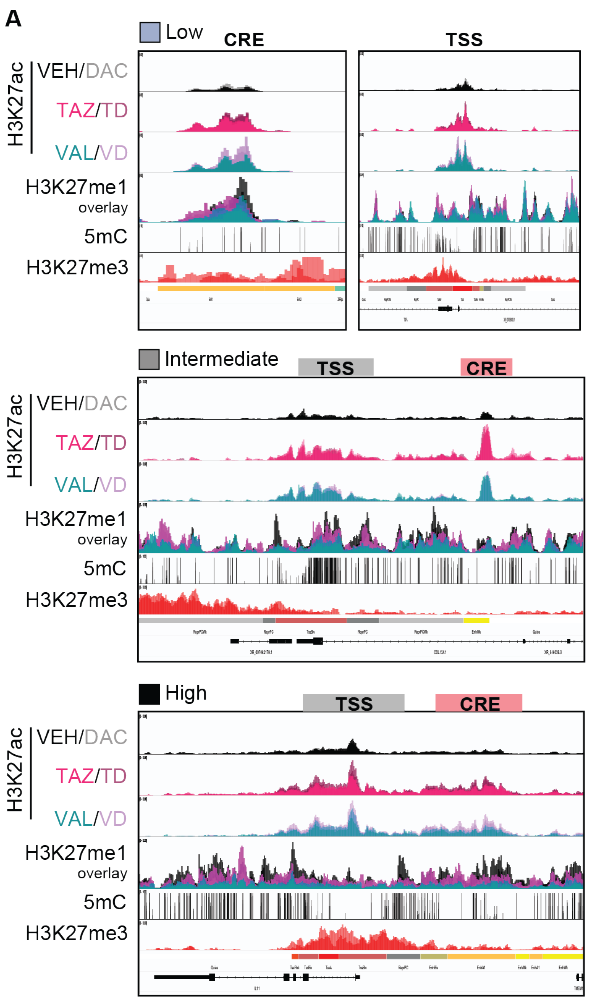

Figure S7. Genome browser shots demonstrating patterns discuss in Figure 5 among the H3K27me1 clusters. Top: Low H3K27me1 cluster, Middle: Intermediate H3K27me1 cluster, Bottom: High H3K27me1 cluster.

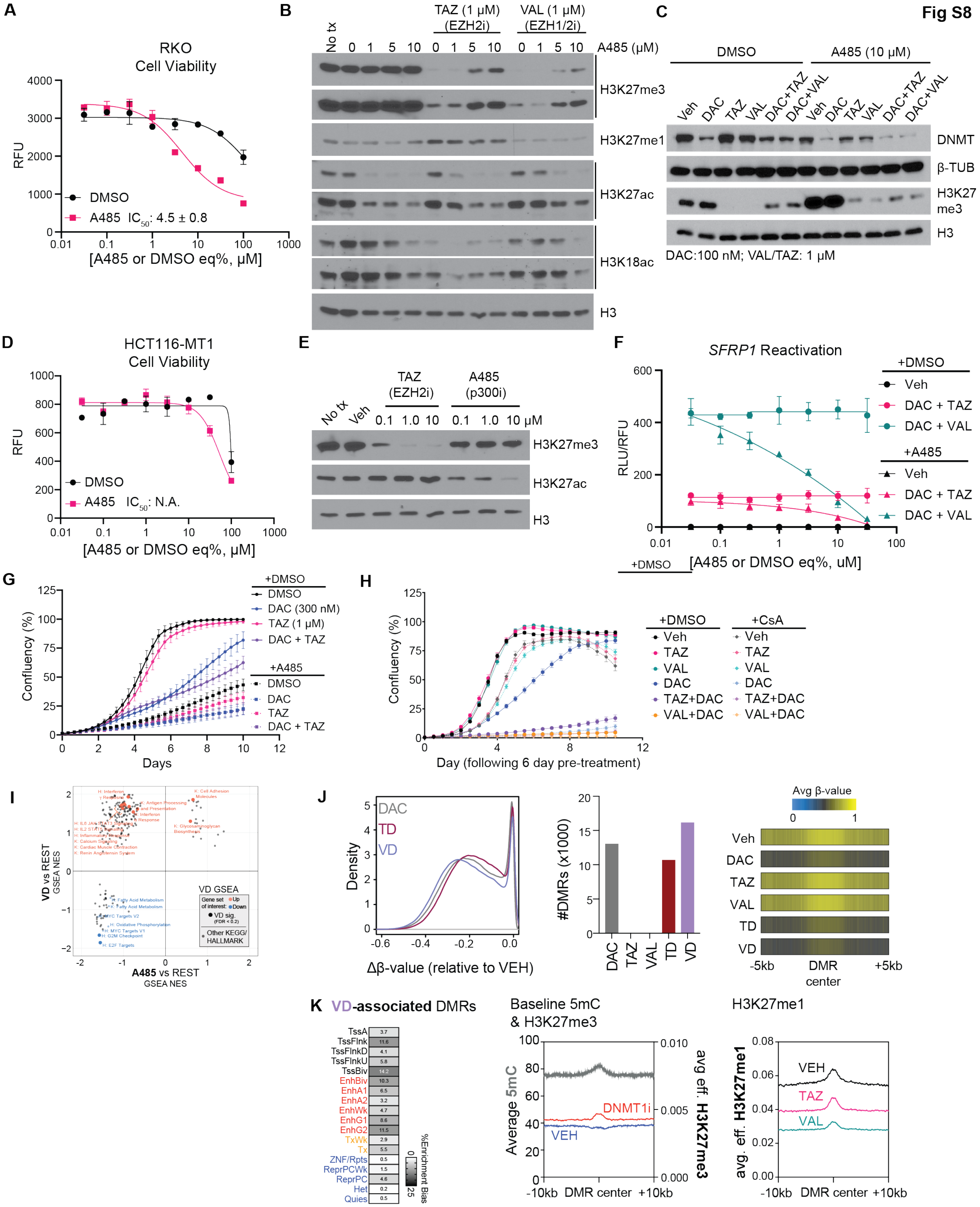

A
RKO
Cell Viability
RFU
DMSO
A485 IC50: 4.5 ± 0.8
[A485 or DMSO eq%, μM]
B
No tx
TAZ (1 μM)
(EZH2i)
VAL (1 μM)
(EZH1/2i)
A485 (μM)
H3K27me3
H3K27me1
H3K27ac
H3K18ac
H3
C
DMSO
A485 (10 μM)
Veh
DAC
TAZ
VAL
DAC+TAZ
DAC+VAL
DNMT
β-TUB
H3K27
me3
H3
DAC:100 nM; VAL/TAZ: 1 μM
D
HCT116-MT1
Cell Viability
RFU
DMSO
A485 IC50: N.A.
[A485 or DMSO eq%, μM]
E
No tx
Veh
TAZ
(EZH2i)
A485
(p300i)
H3K27me3
H3K27ac
H3
F
SFRP1 Reactivation
RLU/RFU
+DMSO
Veh
DAC + TAZ
DAC + VAL
+A485
Veh
DAC + TAZ
DAC + VAL
+DMSO
[A485 or DMSO eq%, uM]
G
Confluency (%)
Days
+DMSO
DMSO
DAC (300 nM)
TAZ (1 μM)
DAC + TAZ
+A485
DMSO
DAC
TAZ
DAC + TAZ
H
Confluency (%)
Day (following 6 day pre-treatment)
+DMSO
Veh
TAZ
VAL
DAC
TAZ+DAC
VAL+DAC
+CsA
Veh
TAZ
VAL
DAC
TAZ+DAC
VAL+DAC
I
VD vs REST
GSEA NES
A485 vs REST
GSEA NES
VD GSEA
Gene set of interest: Up Down
VD sig. (FDR < 0.2)
Other KEGG/ HALLMARK
J
DAC
TD
VD
Density
Δβ-value (relative to VEH)
#DMRs (x1000)
DAC
TAZ
VAL
TD
VD
Avg β-value
Veh
DAC
TAZ
VAL
TD
VD
-5kb
DMR
center
+5kb
K
VD-associated DMRs
TssA 3.7
TssFlnk 11.6
TssFlnkD 4.1
TssFlnkU 5.8
TssBiv 14.2
EnhBiv 10.3
EnhA1 6.5
EnhA2 3.2
EnhWk 4.7
EnhG1 8.6
EnhG2 11.5
TxWk 2.9
Tx 5.5
ZNF/Rpts 0.5
ReprPCWk 1.5
ReprPC 4.6
Het 0.2
Quies 0.5
%Enrichment Bias
Baseline 5mC
& H3K27me3
Average 5mC
avg eff. H3K27me3
DNMT1i
VEH
-10kb DMR center +10kb
H3K27me1
avg. eff. H3K27me1
VEH
TAZ
VAL
-10kb DMR center +10kb

Fig S8

Figure S8. Blocking EZHi/DNMTi-induced transcriptional activation does not prevent anti-proliferation effects

A) Dose response viability curve (CellTiter-Fluor, RFU: relative fluorescence units) for RKO cells exposed to the p300/CBP inhibitor A485 or the equivalent % vehicle (DMSO) for 72 hours. Data are presented as the mean ±SD of technical triplicates.

B) Western blotting for H3K27 and H3K18 modifications in RKO cells exposed to different concentrations of A485 with or without the indicated EZH inhibitors (TAZ: tazemetostat or VAL: valemetostat) at a fixed concentration of 1 μM for 72 hours.

C) Western blotting for H3K27me3 in RKO cells exposed to A485 (10 μM) with or without the indicated EZH inhibitors TAZ or VAL (1 μM) and the DNMTi DAC (100 nM) for 72 hours.

D) Dose response viability curve (CellTiter-Fluor, RFU: relative fluorescence units) for *SFRP1*-NLuc reporter HCT116 cells exposed to A485 or equivalent % vehicle (DMSO) for 72 hours. Data are presented as the mean ±SD of technical triplicates.

E) Western blotting for H3K27 modifications targeted by each molecule in wild-type HCT116 exposed to the indicated TAZ or A485 at different concentrations for 72 hours.

F) NLuc reporter activity measurements following 72-hour treatment with fixed doses of DAC (30 nM) and valemestostat or tazemetostat (VAL or TAZ, 1 μM) combined with a titration of A485 (10 μM). Relative luminescence units (RLU) are normalized to relative fluorescence units (RFU) from the CellTiter-Fluor cell viability assay. Data are mean ± SD of technical triplicates and are representative of three biological replicates.

G) Outgrowth measurements (% confluency) of RKO cells treated once with the indicated inhibitors (DAC, 300 nM and/or tazemetostat [TAZ], 1 μM) with or without A485 (10 μM) and observed for 10 days. Data are the mean ±SEM of technical replicates from a single experiment (n=12 images per timepoint and treatment) and are representative of four biological replicates.

H) Outgrowth measurements (% confluency) of wild-type HCT116 cells treated with the indicated inhibitors (DAC, 30 nM combined with valemetostat [VAL], 1 μM or tazemetostat [TAZ], 1 μM) with or without the calcineurin inhibitor cyclosporin A (CsA, 5 μM) and observed for 10 days. Cells were pretreated with all drugs for two 72-hour cycles prior to replating and treatment at Day 0 of the graph. Data are the mean ±SEM of technical replicates from a single experiment (n=16 images per timepoint and treatment) and are representative of three biological replicates.

I) Scatterplot of GSEA results between VD (VAL+DAC) treatment (y-axis) versus A485 treatment (x-axis). Each dot represents an individual gene set Normalized Enrichment Score (NES) for the designated drug treatment within the HALLMARK/KEGG gene sets.

J) Profiling of DNA hypomethylation induced by EZHi/DNMTi treatment. Left: Density of hypomethylated EPIC probes relative to VEH. Middle: Number of differentially hypomethylated regions (DMRs) for each drug treatment. Right: Average DNA methylation (EPIC array) for VD-associated DMRs across drug treatments.

K) Epigenetic characterization of VD-associated DMRs. Left: Relative enrichment bias across chromHMM states; middle: Average DNA methylation profiles and H3K7me3 signal across drug treatments centered on DMR centers (Gray: baseline DNA methylation, blue: baseline/VEH H3K27me3 signal, red: DNMT1i-induced H3K27me3 signal); and right: Average H3K27me1 signal centered on DMRs.

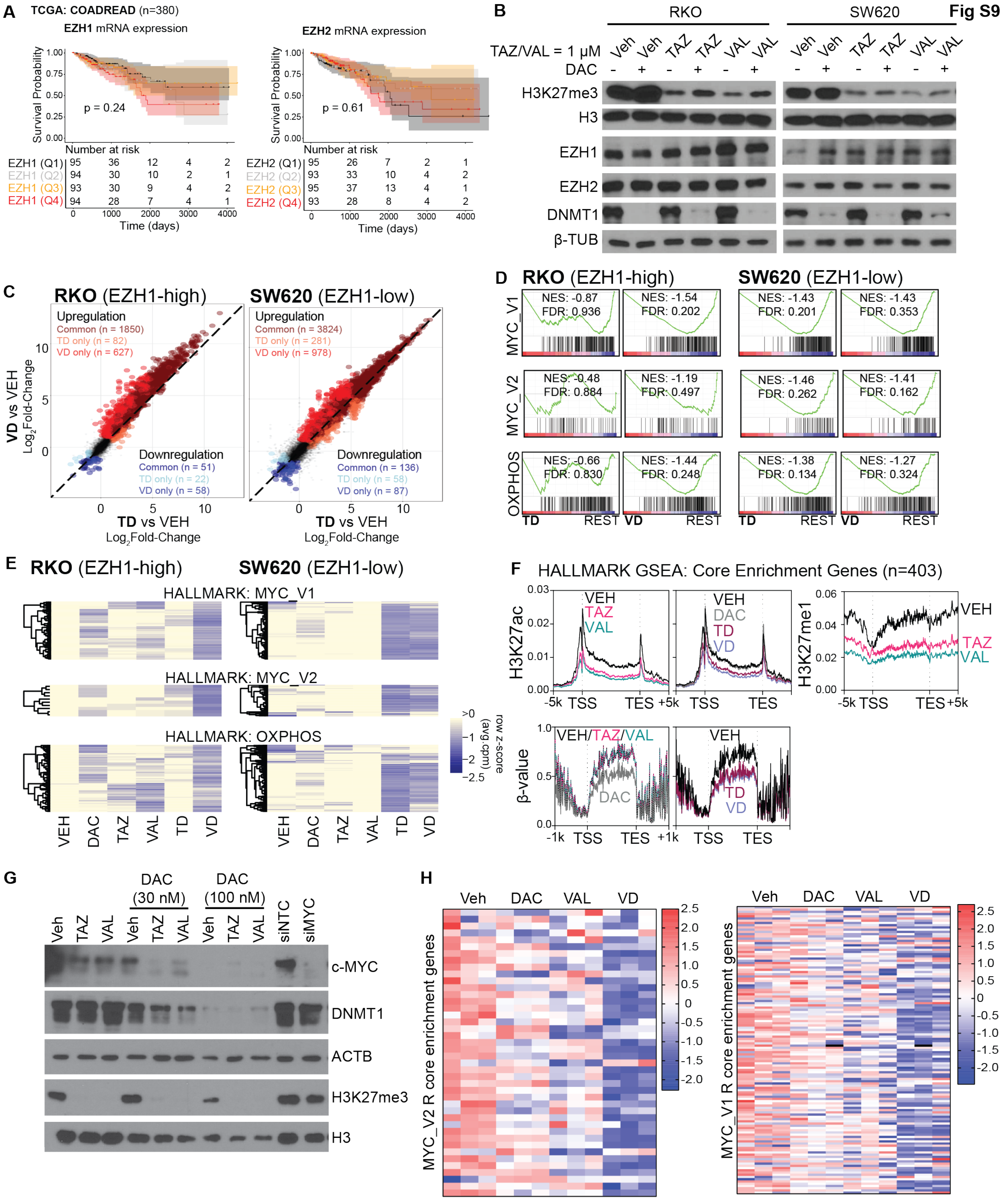

Figure S9. EZH1 predicts sensitivity to EZH inhibitors and downregulation of oncogenic

signaling networks when combined with DNMTi

A) Kaplan-Meier Curves separated by EZH1 (left) and EZH2 (right) mRNA expression for the COADREAD patient cohort. All mRNA expression quartiles were used to calculate significance of overall survival. Shaded areas represent 95% confidence intervals.

B) Western blotting for H3K27me3 levels in RKO or SW620 cells exposed to DAC (300 nM) with or without the TAZ or VAL (1 μM) for 72 hours.

C) Scatterplot of gene expression change (Log2FC relative to VEH) for TD (TAZ+DAC) treatment (x-axis) versus VD (VAL+DAC) treatment (y-axis) in RKO cells (left) and SW620 cells (right). Differentially expressed genes are highlighted by color, and are distinguished by shade as Common, TD only, or VD only.

D) GSEA plots for HALLMARK: MYC Targets V1, MYC Targets V2, and Oxidative Phosphorylation for TD and VD treatments in RKO cells (left) and SW620 cells (right).

E) Heatmaps for gene expression across drug treatment for Core Enrichment Genes in the HALLMARK gene sets described in **D** in RKO cells (left) and SW620 cells (right).

F) Epigenetic profiling of drug treatments at HALLMARK Core Enrichment genes presented in **E** for RKO cells. Left: H3K27ac signal, Right: H3K27me1 signal, and Bottom: 5mC signal.

G) Western blotting for c-MYC levels in wild-type HCT116 cells exposed to DAC (30 or 100 nM) with or without the TAZ or VAL (1 μM) for 72 hours and compared to MYC knockdown with siRNA. NTC: non-targeting control for siRNA.

H) Selected proteins from an untargeted global proteomics experiment in HCT116 cells exposed to DAC (30 nM) with or without VAL (1 μM) for two 72-hour drug treatment cycles (6 days total). Proteins were matched with core enrichment genes from GSEA analysis of HALLMARK MYC_V1 and MYC_V2 Target genes from RNA-seq analysis of HCT116 cells exposed to the same treatment paradigm for one 72-hour cycle. Scale bar is Z-score, each row is an individual protein.
